# Ab Initio Polariton Spectra of ZnTPP Molecules Collectively Coupled Inside an Optical Cavity

**DOI:** 10.1021/jacs.6c01411

**Published:** 2026-05-25

**Authors:** Braden M. Weight, Aaron S. Rury, Yihan Shao, Pengfei Huo

**Affiliations:** † Theoretical Division, 5112 Los Alamos National Laboratory, Los Alamos, New Mexico 87545, United States; ‡ Materials Structural Dynamics Laboratory, Department of Chemistry, 2954 Wayne State University, Detroit, Michigan 48202, United States; § Department of Chemistry, 6187 Brandeis University, Waltham, Massachusetts 02453, United States; ∥ Department of Chemistry, 6927 University of Rochester, Rochester, New York 14627, United States; ⊥ The Institute of Optics, Hajim School of Engineering, University of Rochester, Rochester, New York 14627, United States; # Center for Coherence and Quantum Science, University of Rochester, Rochester, New York 14627, United States

## Abstract

Exciton-polaritons are quasi-particles formed by the quantum mechanical hybridization of electronic and photonic excitations. Despite extensive investigations, a fundamental understanding of molecular polariton spectra and the polariton delocalization from an *ab initio* theoretical perspective remains elusive. We simulate experimentally measured linear transmission spectroscopy of many Zinc­(II) tetraphenylporphyrin (ZnTPP) molecules collectively coupled to a cavity from first principles. Our theoretical approach incorporates many low-lying electronic excitations in ZnTPP molecules, as well as collective light-matter couplings between ZnTPP and the quantized radiation modes, both of which are shown to be the key to accurately recovering the experimental spectra. We further analyzed to what extent the polariton and dark states are delocalized over many molecules, for the first time, using fully *ab initio* descriptions of the molecules. We finally investigate the line width as a function of detuning, providing new theoretical insights into the experimentally observed motional narrowing behavior. Our work presents first-of-its-kind theoretical studies on molecular polariton spectra, offering a new perspective on molecular polariton formation in realistic *ab initio* molecular systems whose rich, many-state nature provides spectral features enabled by the high density of electronic states beyond simple quantum optics models.

## Graphical abstract

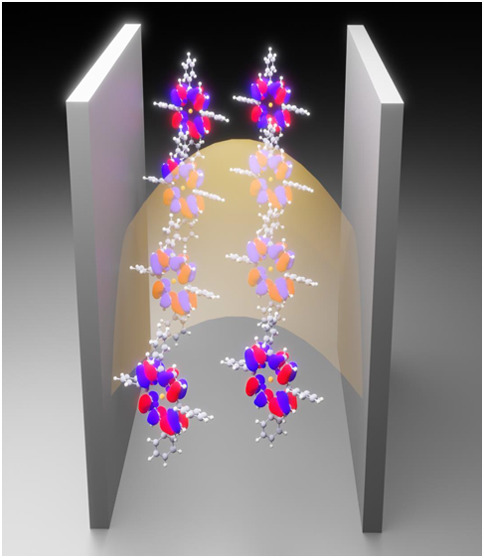

## Introduction

Coupling molecules to the quantized radiation field inside an optical cavity creates a set of new photon-matter hybrid states, called polariton states.^2–7^ These polariton states have delocalized excitations among coupled molecules and the cavity modes. Theoretical investigations play a crucial role in understanding new principles in this emerging field of molecular cavity quantum electrodynamics (QED).^2,3,5,7–21^ Polariton chemistry has been shown to provide potentially new strategies for controlling chemical reactivity^4,13^ and photophysics^14,20–23^ in a general way by manipulating the fundamental properties of photons to enable chemical transformations^13,24^ that can profoundly impact several fields of chemistry, including catalysis and energy production.^25–27^

For *N* identical molecules collectively coupled to one photonic excitation, at the Tavis-Cummings model level of theory, there will be two polariton states, referred to as the upper polariton (UP) and lower polariton (LP), separated by the energy corresponding to the Rabi splitting (Ω_R_). In addition, there are *N* – 1 degenerate dark states, which are destructive linear combinations of molecular exciton states, such that the transition dipole from the ground state to any of these states is zero (optically dark). In the context of realistic systems, molecular disorder (i.e., static and dynamic) plays a pivotal role in resolving the spectroscopic observables of these systems.^21,28^ The linear spectra of molecular polaritons have been extensively investigated, with seminal work from Houdré^29^ using the Tavis-Cummings model^30^ with the explicit consideration of exciton and photonic broadening, laying out the foundational work of explaining polariton lineshapes. Recent investigations^28,31–35^ focused extensively on how various type of disorder influences polariton spectra, including Rabi splitting,^21,28,31^ line width,^36–38^ and the extent of delocalization of polariton and dark states across molecules.^32^ Of particular interest, the recent experiments from Rury on ZnTPP molecules coupled to the Fabry–Perot cavity^1^ show that the experimental line width of polaritons^37,39^ deviates from the theoretical prediction^33,40^ and suggests further motional narrowing behavior.^36,38^ Yet, simple linear response theory based on the Tavis-Cummings model^33,40^ suggests that one should get the results without the above-mentioned additional narrowing (eq 2), and is equivalent to the prediction of the transfer matrix method in classical electrodynamics.^33^ Despite extensive theoretical work on molecular polaritons and linear spectra, there are no *ab initio* investigations that carefully investigate the line shape beyond the typical Tavis-Cummings models. To reconcile the experimental observable with theoretical predictions of quantum optics and quantum electrodynamics, one thus needs to go beyond the simple modes used in quantum optics and adopt an atomistic, *ab initio* description of the molecular polariton system.

In this work, we simulate a recently investigated experimental system^1^ with an ensemble of zinc­(II) tetraphenyl porphyrin (ZnTPP) molecules collectively coupled to an optical cavity, using our previously developed pQED approach.^5,12,13,41^ Our simulated spectra, obtained from first-principles calculations, provide a semiquantitative agreement with the experimental linear transmission spectroscopy.^37^ We compare the theoretical results of collective effects stemming from the number of simultaneously coupled molecules and, importantly, the number of included electronic excitations per molecule. Interestingly, we find that many low-lying electronic excited states per molecule *are required* to reproduce the experimental spectral signatures. This provides a new perspective on the polariton formation in *ab initio* molecular systems whose rich (and complicated) electronic structure may provide additional spectral features enabled by the high density of electronic states in realistic molecules. Finally, we explore the delocalization across the molecular degrees of freedom in the presence of molecular disorder as well as the spectral line width of the upper and lower polariton bands as functions of cavity detuning.

It was widely believed that all linear spectral information for the molecular polariton could be obtained from the transfer-matrix approach, using the frequency-dependent dielectric function of the molecules outside the cavity. In ref 33, it was shown that the line width of the LP polariton will linearly depend on the exciton character |*X*|^2^ (c.f. eq 2). However, recent experimental results in ref 37 and ref 39 suggest that the LP line width has a highly nonlinear dependence on |*X*|^2^, suggesting the breakdown of the simple transfer-matrix approach in terms of providing the correct physical behavior of the LP line width. The ab initio simulations reported in this work allow us to explore this interesting behavior, and our results indeed suggest the same nonlinear behavior of the LP line width with |*X*|^2^ (see Figure 4d), opposed to what had been predicted by the classical transfer matrix method.

Our work highlights the value and necessity of performing *ab initio* simulations of molecular polaritons, which allow us to access properties that are not captured by transfer-matrix calculations. We further theoretically demonstrate that even for the line width of linear polariton spectra, simple transfer-matrix calculations are not sufficient to capture the experimental trend.^37,39^ Finally, beyond the limitation of the transfer matrix approach, these classical approaches do not provide us with microscopic insights into the molecular polaritons. Our *ab initio* approach, on the other hand, provides more fundamental, microscopic insights into the properties of the molecular polariton, providing information on how they depend on collective coupling and light-matter detuning, and guiding the future design principles to manipulate them.

## Results and Discussion

To simulate *ab initio* polaritonic spectroscopy, we solve the Tavis-Cummings Hamiltonian (see schematic representation in Figure 1d) in the dipole gauge under the Born–Oppenheimer and long-wavelength approximations, expressed in eq 4, see Theoretical Methods for details. The collective light-matter coupling is expressed as

1 $$A_{N}=\sqrt{N}A_{0}=\sqrt{N}\cdot \sqrt{\frac{1}{2\omega_{c}\varepsilon V}}$$

where V is the effective mode volume of the cavity, *ε* is the permittivity inside the cavity, and ω_c_ is the cavity frequency. The collective Rabi splitting (at zero light-matter detuning) is $\Omega_{R}\propto A_{N}$ of the optically active polaritonic states. For example, in an ideal Tavis-Cummings model, the Rabi splitting at the resonance condition is $\Omega_{R}=2\omega_{c}\mu_{\mathrm{eg}}\sqrt{N}A_{0}$ for an identical set of *N*, two-level electronic systems all coupled to the cavity resonantly.

**Figure 1:**
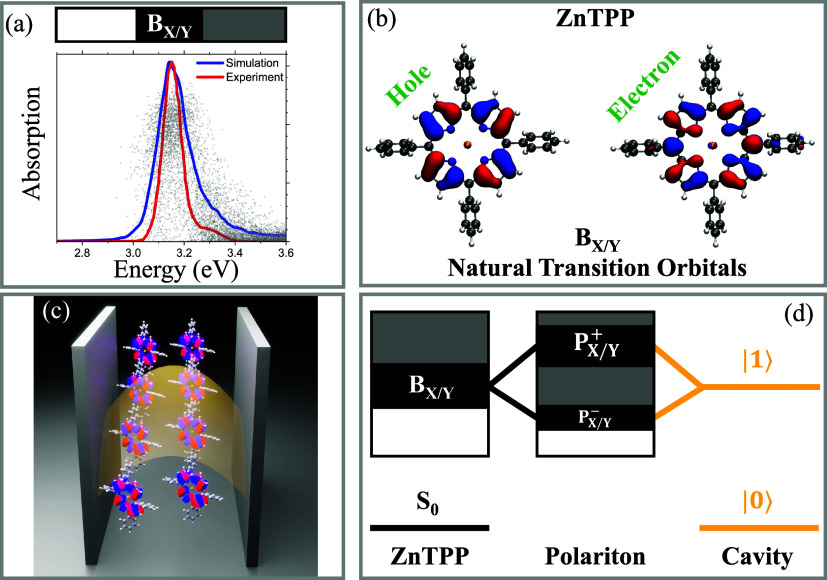
(a) Absorption spectra of the ZnTPP system outside the cavity with experiment (red, reproduced from ref 1) and simulation (blue). The gray dots indicate the oscillator strength of B_X/Y_ state computed at each molecular geometry within an ensemble. (b) The bright transition in the ZnTPP molecules is represented as the dominating natural transition orbitals (NTOs). (c) Schematic of ZnTPP molecules coupled to a Fabry–Perot cavity. (d) Diagram of ZnTPP molecules and a cavity mode hybridizing to form polaritonic states. The B_X/Y_ (black region) indicates a bright set of π-π* transitions within the ZnTPP. The gray region indicates weakly electric-transition-dipole active states, and the white region indicates no transitions within that energy range.

Figure 1a presents the absorption spectra of the ZnTPP molecule outside the cavity, obtained from time-dependent density functional theory (TD-DFT) simulations (blue) compared to the experiments (red), showing a reasonable agreement. Figure 1b shows the natural transition orbitals for the optical transition associated with B_X/Y_. Figure 1c presents the schematic of many molecules collectively coupled inside a Fabry–Perot (FP) cavity. Figure 1d shows a schematic of the bare molecular electronic manifold that couples to the bare cavity, both of which hybridize with photonic excitations to form polariton states. The white regions in Figure 1a,d indicate no excited states, black regions indicate the B_X/Y_ character for the excited states (in both bare and polaritonic cases), and the gray color indicates optically dim states.

It is important to note that vibronic effects are very important in the ZnTPP molecular spectra. While we do not calculate the quantum vibrational spectra (i.e., via Herzberg–Teller-like effects), we incorporate the fully anharmonic effects of the vibrational degrees of freedom at the classical level by sampling a nuclear distribution at finite temperature (see Supporting Information for more details). Thus, we are able to capture the asymmetry of the bare excitonic feature outside the cavity (see Figure 1a). The changes in oscillator strength and energy are evident by examining the distribution of gray dots in Figure 1a, which correspond to the oscillator strength (vertical axis) and energy (horizontal axis) for all nuclear snapshots. Thus, we are able to match the asymmetric experimental spectrum outside the cavity by including vibrational effects.

Figure 2 presents the experimental (black curves) and theoretical polaritonic transmission spectra for the ZnTPP molecule(s) in the Fabry–Perot cavity. The experimental data performed by Rury are reproduced from ref 1., and presented in the left column (panels a, f, k) with black curves. In the experiment, the concentration C of the ZnTPP molecules inside a microcavity was varied (with values indicated in each panel) to control the size of the collective light-matter coupling strength. As the concentration increases, the collective coupling strength $A_{N}\propto \sqrt{N/V}\propto \sqrt{C}$ increases, and thus the Rabi splitting $\Omega_{R}\propto A_{N}$ increases. The experimental Rabi splittings were numerically extracted from the experimental curves reported in ref 1, with Ω_R_ = 102 meV (Figure 2a), Ω_R_ = 131 meV (Figure 2f), and Ω_R_ = 173 meV (Figure 2k), respectively. At smaller concentrations of C=0.5mM (Figure 2a), the upper and lower polaritonic spectral peaks are very close to each other, and are overall symmetrical in both spectral width and splitting about the resonance frequency. With larger concentrations, C=1.0mM (Figure 2f) and C=2.0mM (Figure 2k), the UP and LP splitting increases, and the upper polaritonic peak exhibits an increased broadening compared to the lower polaritonic peak. The lower polaritonic peak’s width is largely unchanged with increasing concentration. Furthermore, the spectral tail at high energy ∼ 3.4 eV becomes more pronounced with increasing concentration. This rich behavior in linear spectra already deviates from the prediction of the line shape from a simple Jaynes-Cummings model^42^ or Tavis-Cummings model,^34,38^ both of which suggest equal line widths for UP and LP under the zero detuning case.^40^

**Figure 2:**
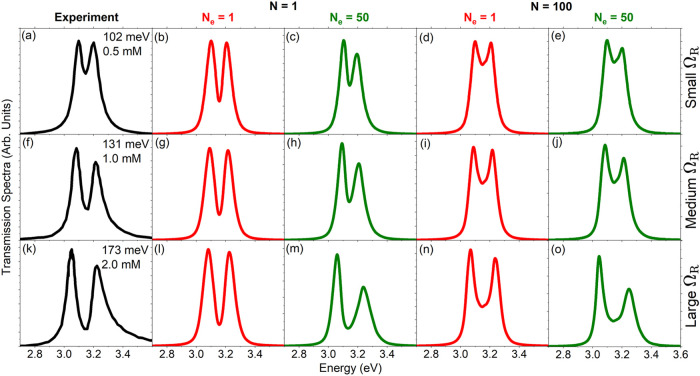
(a, f, k) Experimental spectra (black) reported in ref 1. and simulated polaritonic transmission spectra (red and green) for the ZnTPP molecules coupled inside the cavity. Each simulated column represents a different Hamiltonian: (b, g, l) *N* = 1 and *N*_e_ = 1, (c, h, m) *N* = 1 and *N*_*e*_ = 50, (d, i, n) *N* = 100 and *N*_e_ = 1, (e, j, o) *N* = 100 and *N*_*e*_ = 50. Each simulation is averaged over 1001–*N* snapshots, whose spectra are broadened with a Lorentzian of width σ = 15 meV. The cavity frequency ω_c_ = 3.154 eV is used for all simulated spectra. The collective light-matter coupling strength $A_{N}$ is chosen $\left(A_{N}∼0.006-0.010\;a.u.\right)$ to make the simulated Rabi splitting Ω_R_ close to the experiment value (presented in the first column along with the concentration of ZnTPP).

By numerically solving the polaritonic Hamiltonian (see eq 4), we compute the linear spectra by performing an ensemble average over various thermal realizations of the molecular geometries (see **Theoretical Details** in Supporting Information). We aim to reproduce and understand the spectral features present in the experiment. Columns 2–5 of Figure 2 (colored curves) present the simulated polaritonic transmission spectra (see eq 7) for a range of choices for both the number of molecules *N* and the number of electronic excited states *N*_e_ per molecule. In these cases, we choose the collective coupling strength $A_{N}$ such that the Rabi splitting between the upper and lower polaritonic peaks is roughly the same as the experimental values (presented in the first column of Figure 2). For example, the simulated Rabi splitting for Figure 2b with $A_{N}=0.008$ a.u. produces Ω_R_ = 106 meV, compared to Ω_R_ = 102 meV in experiment Figure 2a.

Figure 2b,g,i present the theoretical simulations of the transmission spectra, using *N* = 1 molecule and including *N*_*e*_ = 1 electronic excited state (the brightest state in the B_X/Y_ region), essentially giving an *ab initio* parametrized version of the Jaynes-Cummings model. This model represents a single two-level system interacting with the cavity mode. Importantly, aside from the matching Rabi splitting between theory and experiment, the simulated spectra shown in these panels do not reproduce the features present in the experimental results (Figure 2a,f,k). Notably, the parametrized Jaynes-Cummings model (Figure 2b,g,i, *N* = 1 & *N*_e_ = 1) misses the broadening of the upper polaritonic feature as well as the spectral tail found in the experimental results (black curves). We propose that this disagreement between simulation and experiment results from the fact that the light-matter Hamiltonian model in Figure 2b,g,i includes only one molecule (*N* = 1) and one electronic excited state per molecule (*N*_e_ = 1). However, the results of simulations considering different parametrizations of the Hamiltonian suggest that the optical spectrum for this molecule is sensitive to the dense manifold of the electronic excited states near the B_*X,Y*_ transitions. As discussed below, the essential photophysics of this polaritonic system appears only when we include this manifold of excited states into the light-matter Hamiltonian.

Including the lowest 50 electronically excited states on each molecule (*N*_e_ = 50) in Figure 2c,h,m immediately yields the asymmetric broadening of the experimental spectra, which is closer to the experimental data, even with only one molecule *N* = 1 in the simulation. Note that asymmetric peak heights for the upper and lower polaritonic features can originate from the choice of the cavity frequency ω_c_, even in an ideal Jaynes-Cummings Hamiltonian, due to finite detuning with the molecular transitions. With a modified Hamiltonian that includes additional nearby electronic states (see Figure 2, columns 2–5), we find that the peak heights and overall profile of the spectra can be modified due to changes in the density of excitonic states included in the choice of Hamiltonian (to be discussed in more detail later). For simplicity, we choose to keep the cavity frequency fixed at ω_c_ = 3.154 eV for all simulations (unless otherwise noted) to examine only the effects of the choice of light-matter model Hamiltonian. We explicitly tested the convergence of the number of included electronic states *N*_e_ on the spectra. See Figure S2 in Supporting Information.

The first key finding of this work is the origin of the asymmetric spectral heights as well as line widths. We assign this observation to the presence of the many optically dim electronic states above B_X/Y_ state. These optically dim, dense manifolds of excitonic states are positively detuned from the cavity transition, yet still couple to the cavity due to their finite transition dipole. In an ideal Hamiltonian, the UP and LP coefficients are |Φ _±_ ⟩∝ *C*_0_^±^ |G, 1⟩ ± *C*_B_XY_,0_^±^ |ψ_B_XY__,0⟩ (see Theoretical Methods below). Due to the additional couplings of the dim manifold, the expansion of the UP is modified as $\left|\Phi_{+}\right\rangle \propto C_{0}^{+}\left|G,1\right\rangle +C_{B_{\mathrm{XY}},0}^{+}\left|\psi_{B_{\mathrm{XY}}},0\right\rangle$ $+\underset{e_{i}\;\neq B_{\mathrm{XY}}\;}{\sum }C_{e_{i},0}^{+}\left|\psi_{e_{i},0}\right\rangle$ , while the LP is largely unmodified from the ideal expansion since there are no additional electronic states below the B_XY_ transition. Therefore, the UP state is broadened due to the added composition of these dim exciton states (see Figure 1d for a schematic illustration). Note that the total photonic character upon integration over the UP feature is largely unchanged, yielding ∼ 0.5 photons, exemplifying that the coefficient of the collective ground state with zero photons is shared between the LP and UP polaritonic states.

The next question one should ask is whether the broadening of the upper polaritonic feature can be explained by the collective coupling between molecules and the cavity mode. In realistic organic molecular polariton experiments,^1,37,39^ the number of molecules collectively coupled to the cavity is at least *N* ≈ 10^6^. In our previous work,^21^ we explored the convergence of linear spectroscopy in the presence of molecular disorder and found that *N* = 100 already provides a robust description of the collective nature of the spectra under strong molecular energy disorder compared to the collective Rabi splitting Ω_R_. In Figure 2d,i,n, we show the simulated transmission spectra for *N* = 100 with *N*_e_ = 1 to selectively demonstrate the effect of collective coupling. This model calculation is essentially based on an *ab initio* parametrized Tavis-Cummings model. This model represents a set of two-level systems interacting with the cavity mode, and the results do not reproduce the asymmetric broadening for the UP states as suggested by the experiments (black).

In Figure 2e,j,o, we use *N* = 100 molecules and include many electronic excited states *N*_*e*_ = 50. Together, this is beyond the typical Jaynes-Cummings and Tavis-Cummings models in quantum optics,^5^ and we believe that this is close to the experimental reality, when both collective light-matter couplings and the manifold of electronic excited states together dictate the polariton photophysics. Indeed, the results successfully reproduce the key experimental features, including the asymmetry of the polariton peak intensities and the further broadening of the UP. Further discussions of these results in Figure 2 are provided in Supporting Information. Note that in our previous work^38^ using exact quantum dynamics simulations and the Holstein-Tavis-Cummings model (with many molecules *N* but only *N*_e_ = 1), and considering dynamical broadening through the Holstein bath model, the UP peaks also exhibits a further broadening compared to those corresponding to the LP, which results from scattering to the dark states manifold that is much less likely to start in the LP state.^38,43^ We have not considered such dynamical scattering to the dark states, and in this work, all the increased broadening of the UP is solely due to the dense manifold of dim electronic excited states. We believe that the experimental reality corresponds to both of these broadening effects, and future work is needed to incorporate both effects simultaneously.

We now focus on a more quantitative understanding of polariton linear spectra by examining the individual polariton states’ contributions to the transmission spectra. Figure 3 shows the polaritonic transmission spectra (black), total polaritonic density of states (DOS, blue), and inverse participation ratio (IPR, red) at two collective light-matter coupling strengths $A_{N}$ = (left column) 0.01 and (right column) 0.02 au for (a–d) *N*_e_ = 1 and (e–h) *N*_*e*_ = 50, both with *N* = 100. Note that, as before, each panel includes all thermal realizations in the ensemble average. For more information on the simulated quantities as well as for additional data on weaker collective light-matter coupling strengths, see eq 7, eq 8, and eq 9 in Theoretical Methods below as well as Figure S3 and Figure S4 in **Theoretical Details** in Supporting Information. Here, the dark states dominate over those of the optically active polariton manifold. The transmission spectra, on the other hand, report the optical brightness and are thus dominated by the upper and lower polariton features (c.f. eq 7 and eq 8).

**Figure 3:**
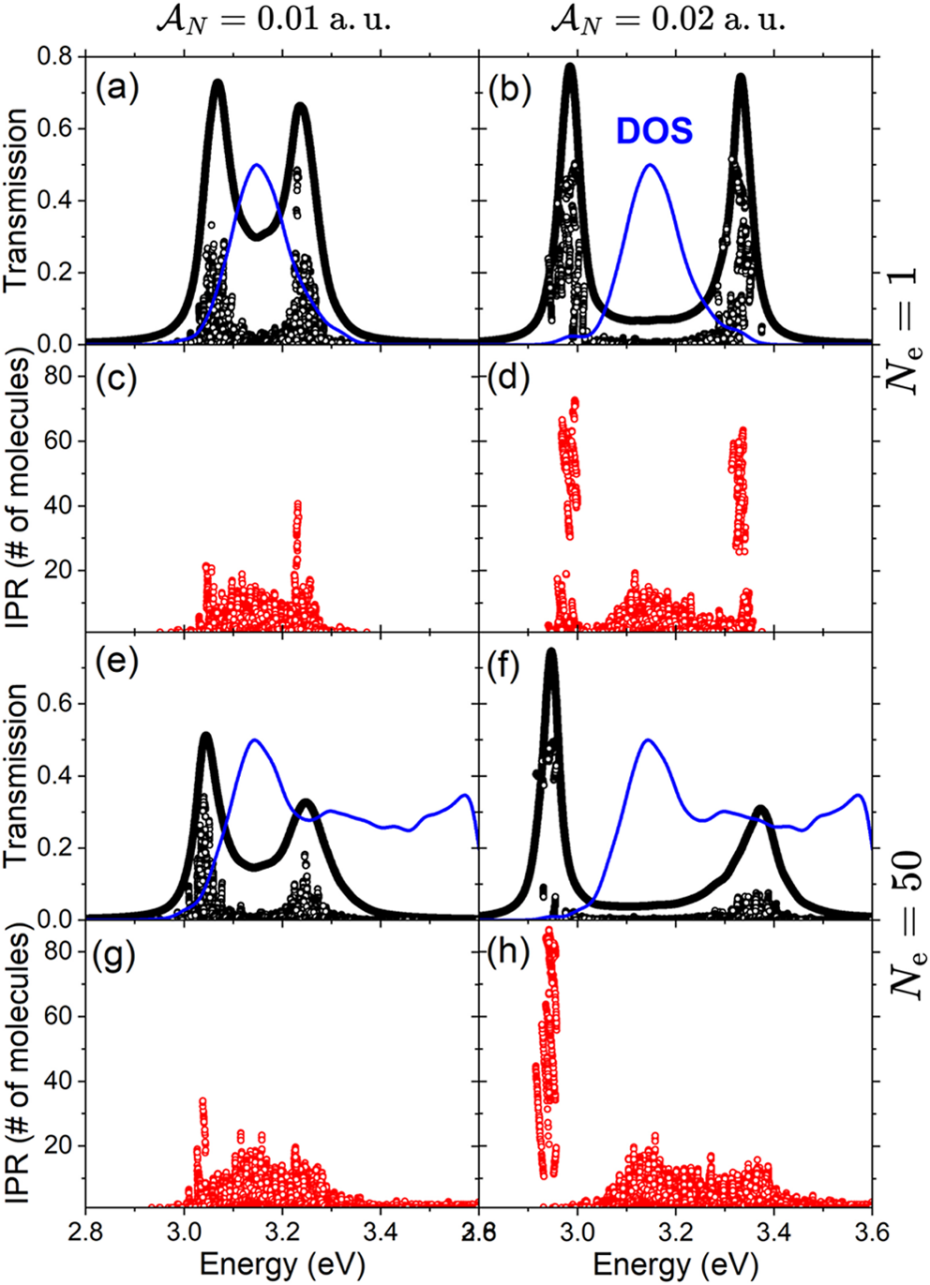
(a,b,e,f) Transmission spectra and (c,d,g,h) inverse participation ratio (IPR) at collective light-matter coupling strengths $A_{N}=0.01$ a.u. (left column), and $A_{N}=0.02$ a.u. (right column). In all panels, the cavity frequency is ω_c_ = 3.154 eV and *N* = 100 molecules with *N*_e_ = 1 (a-d) and *N*_*e*_ = 50 (e-h) electronic states per molecule. The total polaritonic density of states (DOS) is shown in blue alongside the transmission spectra.

The most important difference between the *N*_e_ = 1 and *N*_*e*_ = 50 models is that the upper polariton spectral feature becomes energetically delocalized among the many nearby electronic excited states in that case. The *N*_e_ = 1 case does not capture the feature of sharing photonic character with those nearby states since they are not included in the model. Thus, for the *N*_e_ = 1 case, the maximum transmission intensity of the upper polaritonic feature (black circles in Figure 3b) occurs at *N*_α_ = ⟨Φ_α_ |*â* ^†^ *â* |Φ_α_ ⟩∼ 0.5 for the cases when the transition energies are near resonant with the cavity frequency. On the other hand, for the case of *N*_*e*_ = 50 (Figure 3f), only ⟨Φ_α_ |*â* ^†^ *â* |Φ_α_⟩ ∼ 0.05 is observed due to the many electronic excited states being coupled to the photonic transition. We expect the photonic contribution in each state to decrease as the molecular density of excited states increases, as depicted by the total polaritonic DOS (solid blue curve, Figure 3a,b,e,f).

To better understand how the collective effects play a role in the linear spectra, we compute the inverse participation ratio (IPR, see eq 9) for each polaritonic and dark state. Figure 3c,d and Figure 3g,h show the IPR for *N*_e_ = 1 and *N*_*e*_ = 50, respectively, both with *N* = 100. The value of the IPR indicates the degree of delocalization of a particular polaritonic or dark state across the possible number of molecules. In this work, IPR = 1 implies that the polaritonic state is localized to a single molecule, while IPR = *N* = 100 implies that the polaritonic state is perfectly delocalized across all possible molecules. For the case of *N*_e_ = 1 (Figure 3c,d), which is essentially the Tavis-Cummings Hamiltonian, as the collective light-matter coupling $A_{N}$ increases, we find that the IPR also increases. This trend parallels that of the transmission intensity, indicating that larger light-matter couplings lead to both a larger transmission intensity for the polariton features as the peaks move farther away from the disordered dark states near ω_c_ and, at the same time and for the same reason, allow for a more delocalized state. Further, comparing the case of $A_{N}=0.01$ a.u. (panels a, c) and $A_{N}=0.02$ a.u. (panels b, d), one notices that for the smaller coupling strength, the polariton and dark states are not fully localized due to the presence of the disorder, despite the fact that both have visible Rabi splitting in linear spectra. Additional numerical results computed with varying $A_{N}$ values are provided in the Supporting Information. Our finding is in agreement with the recent work by Liu and Xiong^32^ using Tavis-Cumming Hamiltonian in the vibrational strong coupling regime.

For *N*_*e*_ = 50 (Figure 3g,h), the results are different from the *N*_e_ = 1 Tavis-Cummings model. As the collective light-matter coupling $A_{N}$ increases, only the lower polaritonic band increases in molecular delocalization (increase in IPR). The upper polaritonic transmission band becomes energetically delocalized (as shown in Figure 3e,f) compared to the lower polariton band. As a result, the molecular delocalization (IPR) appears equally as delocalized across its spectral width, as well as across the “dark state” manifold near the cavity frequency ω_c_ = 3.154 eV. Overall, the lower polariton reaches a delocalization of nearly 85 molecules (out of 100) compared to the upper polaritonic band, which only reaches 15–20, equal to or less than the “dark states” manifold, which reaches 20–25. This picture is new compared to previous studies on the Tavis-Cummings model,^29,32^ because in quantum optics models, one usually only considers one electronic excited state, whereas in real molecules, a dense manifold of electronic excited states needs to be considered. One can also explicitly consider the orientational disorder of molecules by assuming an isotropic orientation of molecules inside the cavity. These results are presented in the Supporting Information, and as expected, the isotropic orientation skrinks Rabi Splitting as $\Omega_{R}\cdot \sqrt{\langle \cos\theta \rangle^{2}}∼\Omega_{R}/\sqrt{3}$ .

Finally, we investigate the line width of the UP and LP peaks with the change of light-matter detuning, which has been explored by recent experiments^37,39^ and was explained from the motional narrowing picture.^37–39^ We consider the *N* = 100 case to capture the collective effect, and with *N*_*e*_ = 18 to capture the effect of including many electronic excited states. To reduce the computational cost, we exclude the two lowest excitonic states S_1_ and S_2_ (*E* ∼ 2 eV), which are attributed to the Q-bands of the ZnTPP molecule, as they do not contribute to the spectra. Thus, the included electronic states presented in Figure 4 are S_3_–S_21_. Furthermore, we do not consider dipole orientational disorder effects (as shown in Figure S5 in Supporting Information) due to their insignificant impact on the spectra when including many electronic states (see Figure S5b).

**Figure 4:**
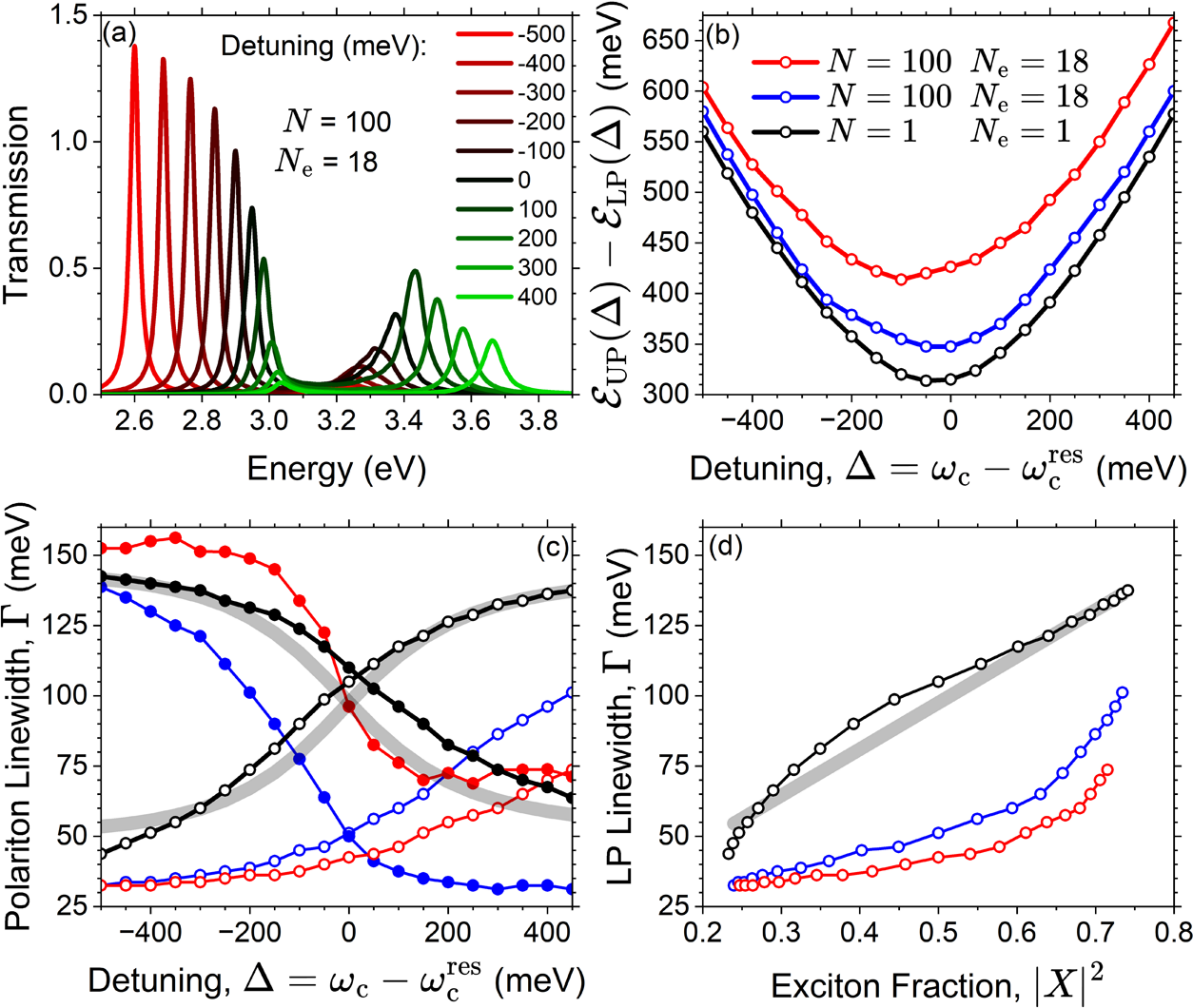
(a) Transmission spectra as a function of the cavity detuning Δ = ω_c_ – ω̅_ex_, whereω̅_ex_ = 3.154 eV is the exciton frequency corresponding to the maximum of the B_X/Y_ molecular absorption feature (see Figure 1a). (b) Energy difference between the upper and lower polaritonic spectral features as a function of the cavity detuning $E_{\mathrm{UP}}\left(\Delta \right)-E_{\mathrm{LP}}\left(\Delta \right)$ . Note that the resonant Rabi splitting Ω_R_ is usually defined at the minimum of these functions and is not preserved between Hamiltonians. (c,d) Polariton broadening Γ (spectral line width) as functions of the (b,c) cavity detuning Δ and (d) exciton fraction |*C*|^2^ for the LP. We show three different theoretical models: *N* = 1, *N*_e_ = 1 (black), *N* = 100, *N*_e_ = 1 (blue), and *N* = 100, *N*_*e*_ = 18 (red). For all panels, the collective light-matter coupling strength is $A_{N}=0.020$ a.u. In panels (c,d), open circles indicate the lower polaritonic (LP) spectral feature, while filled circles indicate the upper polaritonic (UP) feature. The thick gray lines in panels (c,d) indicate no-disorder, two-level model results, based on eq 2.

Figure 4a presents the transmission spectra with respect to the detuning of the cavity frequency Δ = ℏω_c_ – ℏ ω̅_ex_ with respect to the resonance condition of the central excitonic band ℏ ω̅_ex_ = 3.154 eV, with the system of *N* = 100 and *N*_*e*_ = 18. Figure 4b presents the energy difference between the upper and lower polaritonic spectral features as a function of the cavity detuning $E_{\mathrm{UP}}\left(\Delta \right)-E_{\mathrm{LP}}\left(\Delta \right)$ . The three curves shown are for the same system in Figure 4a with *N* = 100 & *N*_*e*_ = 18 (red), as well as two other model Hamiltonians corresponding to *N* = 100 & *N*_*e*_ = 1 (blue, Tavis-Cummings model with disorders) and *N* = 1 & *N*_e_ = 1 (black, Jaynes-Cummings model). Note that the resonant Rabi splitting Ω_R_ is usually defined at the minimum of these functions at an effective resonance condition between molecular and cavity photon energies, which is not preserved between model Hamiltonians.

When including additional electronic states per molecule (Figure 4b, red) or additional molecules for the same number of electronic states (Figure 4b, blue), we find that the Rabi splitting Ω_R_, or more generally the UP/LP peak splitting for all detunings, is always increased due to the additional couplings introduced by either the higher-energy electronic levels or the disordered molecules. When including more molecules while only including *N*_e_ = 1 state (Figure 4b, blue) compared to the Jaynes-Cummings model (*N* = 1 and *N*_*e*_ = 1, black), the Rabi splitting is known to be increased due to the additional static disorders of the exciton frequencies included in the Tavis-Cummings model, which perturbatively enlarge the effective Rabi splitting.^21,28,31^ See Figure S6 in Supporting Information for all cases. Furthermore, for the many-state case *N*_*e*_ = 18 (Figure 4b) the minimum of the Rabi splitting as a function of the detuning is shifted from Δ = 0 eV toward negative detuning Δ ≈ – 100 meV in the presence of many electronic states, indicating that the presence of higher-energy electronic states provides a further lowering of the effective resonance frequency, which has been previously indicated using perturbation theory.^44^

Figure 4c presents the polariton spectral line widths Γ of the upper polariton (filled circles) and lower polariton (open circles) as functions of the detuning of the cavity frequency, obtained from the fwhm value of the simulated spectra. The color codings are the same as those used in Figure 4b. The thick gray curves represent the results of the simple Jaynes-Cummings model (or Tavis-Cummings model with no disorders) with an exciton broadening and photon broadening,^38^ which is identical to the results of transfer matrix simulations through classical electrodynamics (i.e., Maxwell’s Equations).^33,40^ The LP line width is

2 $$\Gamma_{-}=\left|X{|}^{2}\kappa +\right|C{|}^{2}\gamma$$

where γ is the exciton line width, and κ is the cavity line width, |*X*|^2^ = 1 – |*C*|^2^, and the Hopfield coefficient |*C*|^2^ indicates the exciton character of the LP state

3 $$|C{|}^{2}=\frac{1}{2}\left[1+\frac{\Delta }{\sqrt{\Delta^{2}+\Omega_{R}^{2}}}\right]$$

where Ω_R_^2^ = 4*Nω*_c_^2^ *A*_0_^2^μ_eg_^2^ is the Rabi splitting at zero detuning (which is extracted from the simulated spectra). Here, we use an empirical excitonic broadening γ = 180 meV (approximated from Figure 1a) and photonic broadening (due to cavity loss) κ = 15 (matching the spectral broadening used in all previous figures) meV for the best fitting of our Jaynes-Cummings data (black, *N* = 1 and *N*_*e*_ = 1). Note that in a real experimental system, molecular lineshapes exhibit inhomogeneous broadening. As such, using a set of independent two-level systems (based on the JC model) obeying this frequency distribution, then performing an ensemble average will provide a more “appropriate quantum optics limit” of the transfer matrix calculation. This is precisely what our calculation presented in the open black circles in Figure 4d does, where the line shape is obtained upon ensemble average of many independent JC models subject to the frequency static disorders. Nonetheless, the result of these static JC-averaging calculations produces a line shape that closely resembles the analytic answer in eq 2, and deviates from the red and blue open circles that have many *N* molecules in the quantum optics model. The results in Figure 4d highlight the necessity of including many molecules *N* as well as their static disorder in excitation energy (caused by nuclear configuration disorders) in order to capture the essential feature of the nonlinear dependence of LP line width with respect to |*X*|^2^ that has been observed in the experiments.^37,39^

Note that in Figure 1a, each static configuration gives rise to a particular peak, so the broadening parameter σ_ex_ = 15 meV used there accounts for the homogeneous broadening (dynamical disorder), and the inhomogeneous disorders are accounted for by the geometry fluctuations. The empirical parameter γ = 180 meV accounts for the effects of both forms of broadening as it is directly extracted from the simulated linear absorption spectra (Figure 1c). Figure 4d presents the LP line width as a function of the fraction of exciton character |*C*|^2^ (i.e., the Hopfield coefficient), using eq 3. See Figure S6 and Figure S7 in Supporting Information for the transmission, Rabi splitting, and broadening results for additional light-matter Hamiltonians. We emphasize that |*C*|^2^ (eq 3) does not correspond to the actual value of the exciton fraction of the polariton in our simulations (which goes beyond the simple Tavis-Cummings model), but rather it provides an equivalent way to report the Δ dependence of the line width, which is commonly used to interpret the experimental data.^37,39^

At negative detuning Δ < 0, the cavity mode’s frequency is well below the main excitonic excitations. In this case, the lower polaritonic state (open circles) is narrow (Γ ∼ 25–50 meV) for all three models: *N* = 1 & *N*_e_ = 1 (black), *N* = 100 & *N*_e_ = 1 (blue), and *N* = 100 & *N*_*e*_ = 18 (red). This is due to (i) the large photonic character of the LP state (see color codings in Figure 4a) and (ii) the large energetic separation between the lower polaritonic state and the rest of the polaritonic states. Thus, the lower polariton feature is not significantly affected by the molecular disorder, similar to previous discussions at large collective light-matter couplings $A_{N}$ . In contrast, the upper polariton (filled circles) is broadened by ∼3–6 times compared to the lower polariton at large negative detunings (Δ ≈ −500 meV), which is primarily due to the large degree of molecular character in the upper polariton. Furthermore, *N* = 100 & *N*_*e*_ = 18 (red) gives a slightly larger line width for the upper polariton due to the delocalization of the photonic character across higher-energy polaritonic states, even though the total photonic character is small (see Figure 4a, red).

At the near-zero detuning Δ ≈ 0, for the case of the Jaynes-Cummings model *N* = 1 & *N*_e_ = 1 (black), the upper (filled circles) and lower (open circles) polaritonic features are symmetrically broadened (as expected from the theory^38^ gray curve in Figure 4), with Γ ≈ 100 meV. The same is qualitatively true for the Tavis-Cummings model, *N* = 100 and *N*_e_ = 1 (blue), but with a much lower overall broadening of Γ ≈ 50 meV. For *N* = 100 & *N*_*e*_ = 18 (red) at Δ ≈ 0, the lower polaritonic feature (open circles) has nearly the same width as for the *N* = 100 & *N*_e_ = 1 case (blue), while the upper polaritonic band is more than twice as broad due to the interactions with the higher energy, dense manifold of electronic states.

At positive detunings Δ > 0, for the *N* = 1 & *N*_e_ = 1 and *N* = 100 & *N*_e_ = 1 cases, the UP (filled circles) and LP (open circles) line widths switch in their relative magnitudes, due to the fact that UP has more photonic character (narrow) and LP contains excitonic character (broad) at positive detuning Δ > 0 case. The large asymmetry in the *N* = 100 & *N*_e_ = 1 case between the upper polariton at negative detuning and the lower polariton at positive detuning arises due to the asymmetry of the bright exciton under thermal disorder, which manifests under the collective coupling regime. Importantly, for *N* = 100 & *N*_*e*_ = 18, both spectral features converge to the same broadening with increasing cavity detuning Δ, Γ ≈ 70 meV. The upper polariton in positively detuned cases cannot become energetically localized due to the presence of the higher-energy electronic states. As can be seen in Figure 4a (Δ = 400 meV, green), the photonic character of both the upper and lower polaritonic bands is significantly spread among the nearby polaritonic states due to the large density of molecular states in both regions.

In the early experiments,^37,39^ the deviation of the LP line width from the expected results of the Jaynes-Cummings model (or Tavis-Cummings model with no disorders) is observed. The LP line width predicted by the Jaynes-Cummings model (or Tavis-Cummings model without disorders), expressed in eq 2, is illustrated by the gray curve in Figure 4d, which is a linear function of |*C*|^2^. As discussed in the recent theoretical works,^33,40^ eq 2 is equivalent to the transfer matrix calculations (which can be interpreted as an optical filtering effect of the cavity on molecular linear spectra), and requires no quantum QED Hamiltonian diagonalization. Our numerical results with *N* = 1 & *N*_e_ = 1 model (Jaynes-Cummings type) align closely with eq 2. The *N* = 100 & *N*_e_ = 1 model (blue) and *N* = 100 & *N*_*e*_ = 18 model (red) result in an LP line width deviating from the Jaynes-Cummings model (gray). In particular, when considering the Tavis-Cummings model with disorders (blue curve in Figure 4d), the LP line width deviates from eq 2, agreeing with the recent theoretical work on the Kubo-Anderson model^36^ when considering the static disorder limit. On the other hand, within the *N* = 1 & *N*_e_ = 1 (black) and *N* = 100 & *N*_e_ = 1 (blue) models, the upper (filled) and lower (open) polaritonic widths as functions of the exciton fraction follow similar trends in both shape and magnitude. The *N* = 100 & *N*_*e*_ = 18 case, however, does not show the same trend for its LP and UP line width. Instead, the upper polariton resides near the ideal line, but the lower polariton is substantially reduced in width.

In ref 37., this deviation of the LP line width from the expected JC model (so-called the subaverage behavior) was originally interpreted as the motional narrowing behavior. In ref 38., we have used exact quantum dynamics simulations to investigate such behavior and obtained a similar behavior, with a Holstein-Tavis-Cummings model that considers dynamical disorder in the absence of static disorders. In this work, on the other hand, we have seen that this additional narrowing of LP could also originate from either a static disorder in the Tavis-Cummings model, or simply the manifold of the electronic excited states. Our work suggests that the line widths are beyond a simple “optical filtering picture”^33^ predicted by eq 2, at least for the ZnTPP molecular system coupled to the cavity. This suggests that one may need to diagonalize the QED Hamiltonian to obtain the accurate optical spectra of the polariton systems, or at least, consider more accurate molecular response properties in the transfer matrix calculations in the classical electrodynamics simulations.^33,45,46^

## Conclusion

In this work, we report the first *ab initio* polariton spectra simulations with many molecules (*N*) collectively coupled to the cavity, while considering many electronic excited states (*N*_e_), which goes beyond the typical Tavis-Cummings model in quantum optics. Our calculations fully consider all types of disorders, including geometry-fluctuation-induced exciton frequency disorders and dipole angular disorders, while fully capturing the atomistic and *ab initio* details of molecular polaritons. Our theoretical results provide an accurate description of the experimentally measured line shape,^37,39^ including both the line shape and the detuning dependence of line width. We emphasize that the inclusion of many electronic states per molecule in the model light-matter Hamiltonians is essential to gain access to asymmetric features of real molecules.

Our results indicate that the ubiquitously used two-level descriptions in Jaynes-Cummings or Tavis-Cummings models can not capture the detailed physics of linear spectroscopy in experiments of realistic molecular systems, such as ZnTPP. This is because the many nearby electronic states contribute non-negligible effects to the spectra (see Figure 2). One reason is that upon nuclear fluctuations, two or more states can exchange/share character and thus both contribute to the overall light-matter coupling and induce broadening of the spectral feature. In this case, neither state can be neglected. Alternatively, there can be two nearly degenerate (ϵ_*j*_ – ϵ_*k*_ ≈ Ω_R_) electronic states that simultaneously have appreciable transition dipole moments. Both situations are present in the current example of ZnTPP (see Figure 1a). Two-level molecular models, however, should be able to effectively capture small molecules which have well-separated electronic excitation frequencies that are well-beyond the Rabi splitting, ϵ_*j*_ – ϵ_*k*_ ≫ Ω_R_/2 (i.e., atomic-like systems).

Furthermore, we suggest that the number of molecules that are included in the polaritonic Hamiltonian is less important than a proper average over the dynamical/static disorders in the system. This was explicitly shown for model systems in ref 21., where convergence of the spectroscopy was achieved with *N* ∼ 100 molecules. For polariton relaxation dynamics^43^ and decoherence, as well as linear spectra computed from the response function,^34^ it turns out that the polariton dynamics in the Holstein-Tavis-Cummings model are sensitive to the collective quantities $\sqrt{N}A_{0}$ , and are less sensitive to the detailed values of *N* or $A_{0}$ . As shown by the comparison in Figure 2, the shape of the simulated spectra is nearly independent of the number of molecules *N*, and the asymmetric broadening features of the experimental spectra are captured only when *N*_e_ > 1 (i.e., the upper polariton is broadened through interactions with the higher-energy electronic states).

We further explored the delocalization of the polariton wave functions using the inverse participation ratio (IPR), which indicated that the upper and lower polariton features exhibit largely delocalized transitions (Figure 3d). This delocalization increases with increasing Rabi splitting $\Omega_{R}\propto A_{N}$ . When adding additional electronic states from ZnTPP, the upper polariton becomes more spatially localized and energetically delocalized due to the additional interactions (Figure 3h). However, the lower polariton’s delocalization is enhanced by the inclusion of additional electronic states. We also examined the effects of additional angular disorder on the system (Figure S5 in Supporting Information), which showcased a systematic reduction in the Rabi splitting Ω_R_, consistent with previous analytical results. Additionally, we found a reduction in the molecular delocalization for states, which were previously delocalized (70% → 40% delocalization, Figure S5c). The angular disorder exhibited a larger effect on the polaritonic delocalization for idealized systems with *N*_e_ = 1 (Figure S5c) compared to *N*_e_ > 1 (Figure S 5d). Our work brings an *ab initio* picture to the previous seminal work using the Tavis-Cummings model with frequency disorders.^29,32^

Finally, we also explored the effects of detuning on the spectral features (Figure 4), including the Rabi splitting Ω_R_ and the line width Γ of the upper and lower polaritons. Importantly, our *ab initio* results suggest that the Jaynes-Cummings type model (*N* = 1 & *N*_*e*_ = 1) essentially produces the line widths that agree with the analytic model (eq 2), whereas both the Tavis-Cummings type model with disorders (*N* = 100 & *N*_*e*_ = 1) and the many states model (*N* = 100 & *N*_*e*_ = 18) suggest that LP line width will exhibit additional narrowing compared to the analytic model, exhibiting a nonlinear behavior as a function of the Hopfield coefficient |*C*|^2^ (see Figure 4). Our results agree with the experimental observation,^37,39^ indicating the possibility that such narrowing could originate from static disorders and many electronic states, in addition to the originally proposed motional narrowing.^37,38^

We hope our work will inspire continued explorations into the collective effects in light-matter hybrid systems and provide a fundamental, *ab initio* understanding of molecular polariton and its spectra, beyond the existing paradigms of quantum optics models.

## Theoretical Methods

We use the nonrelativistic cavity quantum electrodynamics Hamiltonian under the Born–Oppenheimer approximation and dipole gauge^2,7,47^ to compute the polariton eigenstates and molecular polariton spectra. The total Hamiltonian is expressed as

4 $${Ĥ}_{\mathrm{pl}}\left(R\right)=\sum_{n=1}^{N}{Ĥ}_{\mathrm{el}}\left(R_{n}\right)+\hbar \omega_{c}\left({â}^{†}â+\frac{1}{2}\right)+\omega_{c}A_{0}\sum_{n=1}^{N}\left(\mathrm{\mu ̂}\left(R_{n}\right)\cdot ê\right)\left({â}^{†}+â\right)$$

where *Ĥ*_el_(**R**_*n*_) is the electronic Hamiltonian for the *n*_th_ molecule, *â* (*â* ^†^) is the annihilation (creation) operator for the cavity mode, ***ê*** is the cavity polarization vector, **μ̂**(**R**_*n*_) is the dipole operator for molecule *n*. The third term in eq 4 is the light-matter interaction. For the results presented in the main text, we did not consider the dipole orientational disorder, which gives ⟨*i*|**μ̂**|*j*⟩(**R**_*n*_) · **ê** = |**μ**_*ij*_|cosθ_*n*_, with ⟨ cos^2^θ⟩= 1/3 for an isotropic orientational disorder,^29^ and the Rabi splitting will shrink by $1/\sqrt{3}$ . In the Supporting, we present the results that do explicitly include these disorders (Figure S4), and confirmed that the role of this type of disorder is just to reduce the Rabi splitting, equivalent to changing *A*_0_. Becasue we are working with a much larger *A*_0_ and much smaller *N* compared to the experiments, we did not include the angle disorder in the main text, but we do provide additional results that do have them in the Supporting Information (see Figure S4).

The electronic eigenvalue equation for each molecule *n* is *Ĥ*_el_(**R**_*n*_) |ψ_*j*_(**R**_*n*_)⟩= ϵ_*j*_(**R**_*n*_)|ψ_*j*_(**R**_*n*_)⟩, can be can be solved in parallel across all molecules by any electronic structure approach, generating adiabatic electronic potential energy surfaces ϵ_*j*_(**R**_*n*_) for molecule *n* and adiabatic state |ψ_*j*_(**R**_*n*_)⟩. We denote *j* = 0 as the ground state for a given molecule *n*, which is |ψ_0_(**R**_*n*_)⟩. For *j* ∈ [1, *N*_*e*_], |ψ_*j*_(**R**_*n*_)⟩ represents the electronic excited states. The photonic Hamiltonian, a quantum harmonic oscillator, can be solved analytically ${Ĥ}_{\mathrm{ph}}\left|k\right\rangle =\hbar \omega \left(k+\frac{1}{2}\right)\left|k\right\rangle$ . The polariton eigenvalue problem can be formally written as

$${Ĥ}_{\mathrm{pl}}\left(R\right)\left|\Phi_{\alpha }\left(R\right)\right\rangle =E_{\alpha }\left(R\right)\left|\Phi_{\alpha }\left(R\right)\right\rangle$$ 5

where $E_{\alpha }\left(R\right)$ are the polaritonic potential energy surfaces and |Φ_α_(**R**)⟩ are the adiabatic polaritonic states, expanded using

6 $$\left|\Phi_{\alpha }\left(R\right)\right\rangle =C_{0}^{\alpha }{⊗}_{n}\left|\psi_{0}\left(R_{n}\right)\right\rangle ⊗\left|1\right\rangle +\sum_{n=1}^{N}\sum_{j=1}^{N_{e}}C_{\mathrm{jn}}^{\alpha }{⊗}_{m\neq n}\left|\psi_{0}\left(R_{m}\right)\right\rangle ⊗\left|\psi_{j}\left(R_{n}\right)\right\rangle ⊗\left|0\right\rangle$$

where we have restricted to the single excitation subspace (where either one molecule or the cavity mode can be excited, but not restricting which excited state on molecule *n*). This approximation is valid under the assumption that the single-molecule light-matter coupling *A*_0_ is small and where the energy range of interest is near the fundamental resonance between the molecular and photonic frequencies, ω ≈ ϵ_1_^(*n*)^ – ϵ_0_^(*n*)^ (i.e., far from the collective ground state and doubly excited configurations’ energy). Further, the expansion coefficients *C*_0_^α^ (**R**) and *C*_*jn*_^α^(**R**) parametrically depend on the nuclear configurations **R** ∈ {**R**_1_, ...**R**_*n*_, ...**R**_*N*_}. Both *C*_0_^α^ and *C*_*jn*_^α^ as well as $E_{\alpha }\left(R\right)$ are obtained by directly diagonalizing the matrix of *Ĥ*_pl_(**R**) using the basis indicated in eq 6. We explicitly tested the convergence of the number of included electronic states *N*_e_ on the spectra. See Figure S2 in Supporting Information.

The linear transmission spectra are computed as

7 $$T\left(\omega \right)={\left\langle \sum_{\alpha }N_{\alpha }\cdot \delta \left(\hbar \omega -E_{\alpha }\left(R\right)\right)\right\rangle }_{R}\approx {\left\langle \frac{\sigma }{\pi }\sum_{\alpha }\frac{|C_{0}^{\alpha }\left(R_{n}\right){|}^{2}}{{\left(\hbar \omega -E_{\alpha }\left(R\right)\right)}^{2}+\sigma^{2}}\right\rangle }_{R}$$

where $N_{\alpha }=\left\langle \Phi_{\alpha }\left(R\right)\right|{â}^{†}â\left|\Phi_{\alpha }\left(R\right)\right\rangle$ is the photon number expectation value under state |Φ_α_(**R**)⟩, σ = 15 meV is the finite-width Lorentzian broadening parameter representing the broadening contribution from both exciton decay (dynamical contribution) and photonic decay, and the ensemble average ⟨···⟩_**R**_ represents an average over geometries sampled from the Born–Oppenheimer MD simulations. The detailed value of the parameter is provided in each figure and tested in Figure S1 in Supporting Information. Similarly, the total polariton density of states (DOS) is computed as

$$\mathrm{DOS}\left(\omega \right)={\left\langle \underset{\alpha }{\sum }\delta \left(\hbar \omega -E_{\alpha }\left(R\right)\right)\right\rangle }_{R}\approx {\left\langle \frac{\sigma }{\pi }\underset{\alpha }{\sum }\frac{1}{{\left(\hbar \omega -E_{\alpha }\left(R\right)\right)}^{2}+\sigma^{2}}\right\rangle }_{R}$$ 8

The inverse participation ratio (IPR), the molecular delocalization extent on the *a*_th_ polaritonic state, is defined as

$${\mathrm{IPR}}_{a}=\frac{1}{\underset{n}{\sum }{\left(P_{n}^{\alpha }\right)}^{2}},P_{n}^{\alpha }=\frac{\underset{j_{n}\;}{\sum }|C_{j_{n}}^{\alpha }{|}^{2}}{\underset{m,k_{m}\;}{\sum }|C_{k_{m}}^{\alpha }{|}^{2}}$$ 9

where *P*_*n*_^*α*^ is the probability for a polariton state |Φ_α_⟩ to reside on molecule *n*, and *C*_*j*_*n*__^*α*^ is the expansion coefficient of the *j*_th_ electronic molecular excitation on the *n*_th_ molecule for the *α*_th_ polaritonic state (see eq 6).

## Computational Details

The primary electronic transitions are a pair of degenerate states denoted as B_X/Y_, whose hole and electron natural transition orbitals are shown in Figure 1d. The higher-lying, optically dim states (i.e., gray region in Figure 1d) have pure electronic excitation character, as we do not include vibrational Herzberg–Teller effects. Vibrationally resolved exciton-polariton spectra will be the topic of future work. Importantly, these optically dim states still have a finite transition dipole from the ground state. This will play a key role in all the results presented in this work. The transition dipoles of these gray states drastically vary in their intensities as a function of the nuclear coordinates, resulting in a range between 50% and 1% of the maximum B_X/Y_ intensity. Notably, even the intensity of the B_X/Y_ transition (i.e., black region) varies with the nuclear coordinates, sharing its character (and transition dipole strength) with these nearby states. Throughout this work, we choose a cavity frequency that corresponds to the B_X/Y_ electronic transition (see Figure 1d).

We performed Born–Oppenheimer molecular dynamics in the ground electronic state of a ZnTPP molecule, at the level of the semiempirical AM1 Hamiltonian. We used a Langevin thermostat at *T* = 300 K to thermalize the ground state geometries. We sampled a geometry from the dynamics every ∼ 45 fs, yielding 1001 geometries. We use linear-response time-dependent density functional theory (LR-TD-DFT) at the level of B3LYP/6–31G* for each geometry to obtain excited states and the dipole matrix elements. The excitation energies and oscillator strengths of the first 50 singlet excitations were used to generate a thermally averaged molecular absorption spectrum for each molecule. Details are provided in the Supporting Information.

## Supplementary Material



## Notes

Computational details for the spectral intensities, including the thermal average, as well as additional numerical results with a variety of light-matter regimes (PDF)
