# Supplementary material for: Ab Initio Polariton Spectra of ZnTPP Molecules Collectively Coupled Inside an Optical Cavity
Source: J Am Chem Soc. 2026 May 25;148(21):21646–56. doi: 10.1021/jacs.6c01411 (PMC13244444; doi:10.1021/jacs.6c01411)
Supplement: Supplementary file 1 [file ja6c01411_si_001.pdf]

Supporting Information: Ab Initio Polariton Spectra of ZnTPP Molecules Collectively Coupled inside an Optical Cavity

Braden M. Weight,[*,†] Aaron S. Rury,[‡] Yihan Shao,[¶] and Pengfei Huo[*,§,‖,⊥]

†*Theoretical Division, Los Alamos National Laboratory, Los Alamos, NM 87545, U.S.A.*

‡*Department of Chemistry, Wayne State University, Detroit, MI 48202, U.S.A.*

¶*Department of Chemistry, Brandeis University, Waltham, MA 02453, U.S.A.*

§*Department of Chemistry, University of Rochester, Rochester, NY 14627, U.S.A.*

‖*The Institute of Optics, Hajim School of Engineering, University of Rochester, Rochester, NY 14627, U.S.A.*

⊥*Center for Coherence and Quantum Science, University of Rochester, Rochester, New York 14627, U.S.A.*

E-mail: braden.m.weight@lanl.gov; pengfei.huo@rochester.edu

Theoretical Details

We simulated 50 ps of Born-Oppenheimer molecular dynamics in the canonical ensemble (*i.e.*, constant NVT, with T = 300 K) using Langevin dynamics, as implemented in the SQD code.[1] We use AM1[2] for the ZnTPP ground state electronic structure, as implemented in the Gaussian16 package.[3] Discarding the first 1.0 ps for equilibration, we sampled geometries roughly every ∼45 fs, leading to 1001 snapshots. Using the snapshots from the NVT dynamics, we then simulated linear-response time-dependent density functional theory (TD-DFT) as implemented by Gaussian16.[3] Here, we used the B3LYP/6-31G* functional and basis. We then extracted the ground and excited state energies and transition dipole matrix (computed by Multiwfn[4]) for each geometry. The

excitation energies and oscillator strengths of the first 50 singlet excitations were used to generate a thermally averaged molecular absorption spectrum for each molecule.

In previous works, for a single mode, we have expanded the polaritonic wavefunctions as a tensor product of electronic and photonic bases as $|\Phi_\alpha(\mathbf{R})\rangle = \bigotimes_n \sum_{j_n,v} C^{\alpha}_{j_n,v}|\psi_{j_n}(\mathbf{R})\rangle \otimes |v\rangle$. However, in order to extend toward many molecules, we continue to restrict ourselves to a single photonic mode and to the hybrid basis which includes only the collective ground configuration, $|\psi_0, \cdots, \psi_0, 0\rangle$, and the singly excited configurations, $|\psi_0, \ldots, \psi_{j_n \neq 0}, \cdots, \psi_0, 0\rangle$ or $|\psi_0, \ldots, \psi_0, 1\rangle$, of the total Hilbert space defined by the tensor product. This approximation is valid under the assumption that the single-molecule coupling $A_0 = \mathcal{A}_N/\sqrt{N} \propto \Omega_\mathrm{R}/\sqrt{N}$ is small and where the energy range of interest is near the fundamental resonance between the molecular and photonic frequencies, $\omega \approx \epsilon_1^{(A)} - \epsilon_0^{(A)}$ (*i.e.* far from the collective ground state and doubly excited configurations' energy).

The simulated bare molecular absorption spectra (see Fig. 1a of the main text) were simulated by as,

$$\begin{aligned}\mathcal{A}(\omega) &= \Big\langle \sum_n^N \sum_{j_n}^{N_\mathrm{e}} h_{0_n,j_n}(\mathbf{R}_n)) \cdot \delta(\hbar\omega - \epsilon_{0,j_n}(\mathbf{R}_n))\Big\rangle_{\mathbf{R}} \\ &\approx \Big\langle \frac{\sigma}{\pi} \sum_n^N \sum_{j_n}^{N_\mathrm{e}} \frac{h_{0_n,j_n}(\mathbf{R}_n)}{(\hbar\omega - \epsilon_{0,j_n}(\mathbf{R}_n))^2 + \sigma^2} \Big\rangle_{\mathbf{R}}\end{aligned} \tag{S1}$$

where $\epsilon_{j_n}(\mathbf{R}_n)$ is the adiabatic transition energy of the j_th electronic state of the n_th molecule. The unpolarized oscillator strength is defined as,

$$h_{0_n j_n}(\mathbf{R}_n) = \frac{2}{3}\big[\epsilon_{j_n}(\mathbf{R}_n) - \epsilon_{0_n}(\mathbf{R}_n)\big]\, |\mu_{0_n j_n}(\mathbf{R}_n)|^2. \tag{S2}$$

The inverse participation ratio (IPR), the molecular declocalization extent on the α_th polaritonic state, is defined in Eq. 9 in the **Main Text**, denoted as $(\mathrm{IPR})_\alpha(\mathbf{R}) \equiv (\mathrm{IPR})_\alpha$. The delocalization density, plotted in Fig. S5c,d in the **Main Text**, was computed as

$$\mathrm{IPR}(E) = \frac{\sigma}{\pi} \sum_\alpha \frac{(\mathrm{IPR})_\alpha}{(E - \mathcal{E}_\alpha(\mathbf{R}_n))^2 + \sigma^2}. \tag{S3}$$

$$\mathrm{IPR}(\omega) = \Big\langle \sum_\alpha (\mathrm{IPR})_\alpha \cdot \delta(\hbar\omega - \mathcal{E}_\alpha(\mathbf{R})) \Big\rangle_{\mathbf{R}} \approx \Big\langle \frac{\sigma}{\pi} \sum_\alpha \frac{(\mathrm{IPR})_\alpha}{(\hbar\omega - \mathcal{E}_\alpha(\mathbf{R}))^2 + \sigma^2} \Big\rangle_{\mathbf{R}} \tag{S4}$$

Due to the approximations of the underlying electronic structure approach, the excitation energy of the simulated spectra does not exactly align with the experimental data. To make a fair comparison between theory and experiment, we have shifted the experimental data, which only appears in Figs. 1 and 2 in the main text, to match the central peak location provided by the choice of electronic structure, TD-B3LYP/6-31G*. The simulated spectra are blue-shifted compared to the experiment. This shift is constant ($\Delta E_{\mathrm{exp}} = 0.225$ eV) throughout and does not affect the conclusions of this work.

It is important to note that the pQED approach is expected to provide increased efficiency compared to the scQED approaches in the many-molecule case. This is because each complicated molecular DOF – itself a many-electron problem – can be calculated independently (as a separate and parallelizable standard electronic structure problem) before combining with the photonic DOFs and all other precalculated molecular DOFs. Thus, the scaling s of the hybrid Hamiltonian is reduced from, for example, using a scQED-configuration interaction singles (CIS) approach and a single cavity mode, $s_{\mathrm{scQED-CIS}} \sim (N N_{\mathrm{o\to v/mol}} N_{\mathrm{F}})^4$ for scQED-CIS to $s_{\mathrm{pQED-CIS}} \sim N N^4_{\mathrm{o\to v/mol}}$ (Individual Molecules) $+(N N_{\mathrm{e}} + N_{\mathrm{F}})^3$ (Molecule-Photon Interaction) for the equivalent two-step, pQED-CIS approach. Here, $N_{\mathrm{o\to v/mol}}$ are the number of occupied-to-virtual transitions per molecule. It is expected that $s_{\mathrm{pQED-CIS}} \ll s_{\mathrm{scQED-CIS}}$ for all cases in which there are many molecules coupled to the cavity, each with weak single-molecule couplings, and when the photon-induced electron-electron correlation (dominated by the DSE term) is weak. The DSE term dominates the cavity-induced ground state modifications at large single-molecule couplings.[5–9]

It should be noted that Eq. 4 ignores all inter- and intra-molecular interactions among the molecules (*i.e.*, the photon-mediated electron-electron interactions) mediated by the dipole self-energy (DSE) terms.[10–16] Thus, only the direct interactions between the light and matter are included, which are the dominating contributions at weak single-molecule couplings. The DSE term dominates the cavity-induced ground state modifications at large single-molecule couplings.[5–9,17–19]

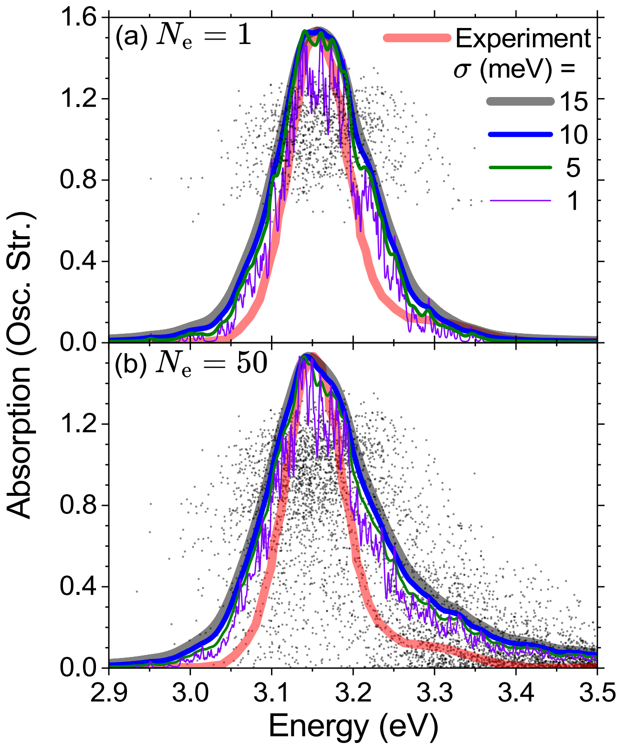

Figure S1: Molecular absorption spectra outside the cavity with various broadening parameters: $\sigma = 15$ meV (grey), $\sigma = 10$ meV (blue), $\sigma = 5$ meV (green), and 1 meV (purple) for (a) $N_{el} = 1$ and (b) $N_{el} = 50$ compared to experimental[20] absorption spectra (red). The small black symbols indicate the absorption intensities (*i.e.*, oscillator strengths) used during the finite-width Lorentzian convolutions. The expected error stemming from the finite-width broadening parameter is $\sim \frac{\sigma}{2}$. In the limit of $N_{ave} \to \infty$, $\sigma \to 0$.

Fig. S1 presents the results of the outside cavity absorption spectra for the ZnTPP molecules at various finite-width Lorentzian broadening parameters $\sigma = 1$ (purple), 5 (green), 10 (blue), and 15 meV (grey) for (Fig. S1a) $N_e = 1$ and (Fig. S1b) $N_e = 50$. The experimental spectra[20] are shown in red. For the $N_e = 1$ case, the distribution of the oscillator strengths (small black symbols) for the bright transition is nearly uniform in intensity as well as symmetric in their distribution. As such, the finite-width Lorentzian convolutions of those oscillators result in highly symmetric Lorentzian distributions. In contrast, the $N_e = 50$ case exhibits largely asymmetric oscillators that vary drastically in intensity. Thus, the finite-width convolutions are also asymmetric.

In varying the magnitude of the finite width σ, the function can be "tuned" to provide a smooth curve. Given infinite numbers of snapshots (oscillators) in which to average the spectra, the width of the convolution needed to provide a smooth curve decreases to zero, $\sigma \to 0$. Thus, the spectra

converge to an intrinsic shape, independent of the choice of broadening function, *e.g.*, Lorentzian, Gaussian, *etc.* In the current work, we have only 1001 snapshots, so our width must also be finite. For both cases of N_e, we show four widths. The smoothest curve is produced at $\sigma = 15$ meV, while $\sigma = 1$ meV provides a highly oscillatory curve not suitable for comparison to experiment. We note that any choice of width retains the necessary physics and does not change any conclusions of this work. This is evidenced by the (i) broadening of the central peak between $N_\mathrm{e} = 1$ and $N_\mathrm{e} = 50$ and (ii) the right-handed tail appearing in $N_\mathrm{e} = 50$. All choices of σ are able to capture these effects.

Figure S2 presents the explicit convergence of the N_e parameter on the spectra for two light-matter coupling strengths $A_0 =$ (Figure S2a) 0.01 and (Figure S2b) 0.02 a.u.. Figure S2c shows the error $= \sum_i |\mathrm{TM}(E_i, N_\mathrm{e}) - \mathrm{TM}(E_i, N_\mathrm{e} = 10)|$. Here in Figure S2, in Figure 4 in the Main Text, and in Figure S5, for convenience, we do not include the two Q-band excitons, which are significantly lower in energy ($\sim$ 2 eV). These excitons never contribute to the spectra because the lower polariton is always more than 0.5 eV away from the Q-band states, even at the strongest coupling strength explored in this work. Thus, in reference to Table S1, we start the convergence from S_3. For clarification, Figure 1a in the Main Text, Figure 2 in the Main Text, and Figure 3 in the Main Text include the Q-band excitons for completeness. Note that the cost of each calculation scales as $(NN_\mathrm{e})^3$, so the $N_\mathrm{e} = 10$ simulation was $\sim 10^3$ more expensive than the $N_\mathrm{e} = 1$ calculation. For perspective, the $N_\mathrm{e} = 1$ simulation takes a few seconds. In terms of the convergence of the spectra, after $N_\mathrm{e} > 5$, the spectra show very little or no visual difference.

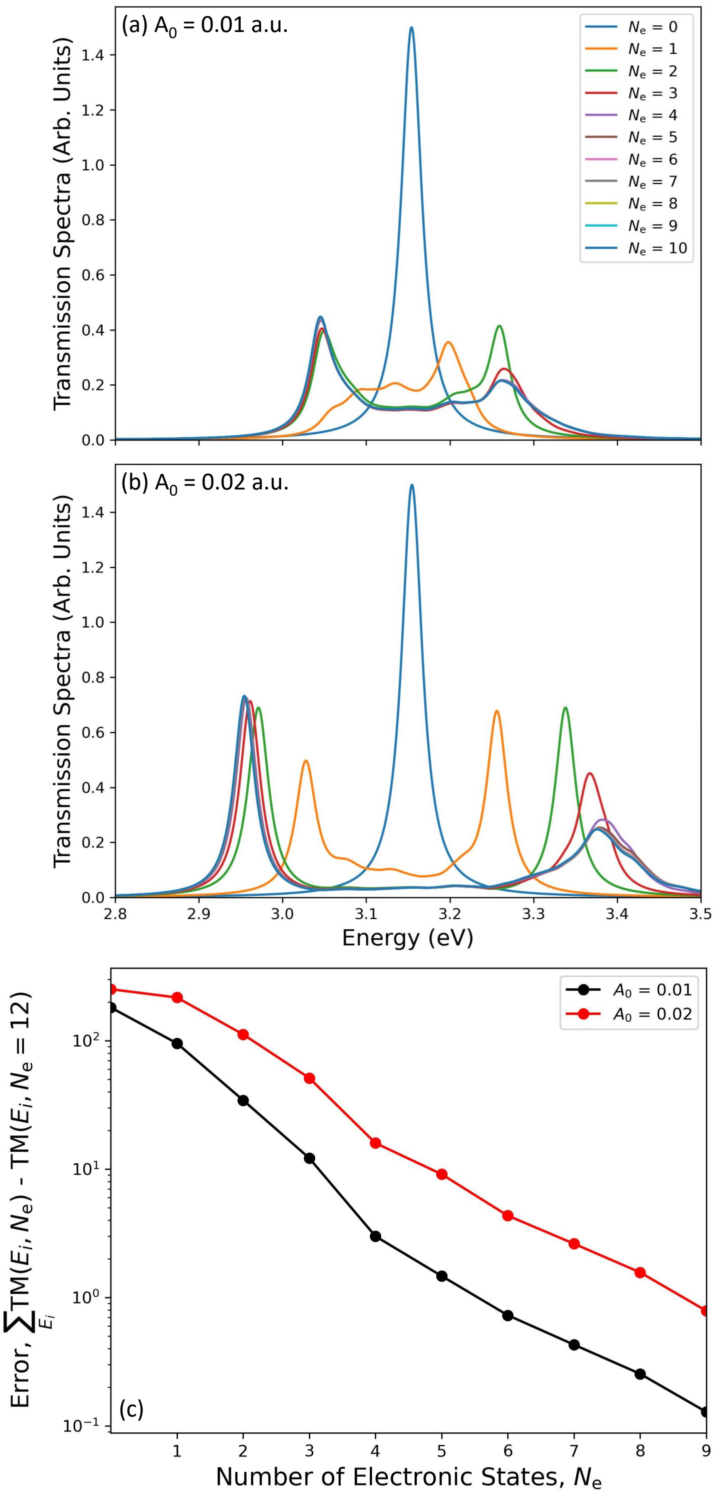

Figure S2: Convergence of the spectra at (a) $A_0 = 0.01$ a.u. and (b) $A_0 = 0.02$ for N_e ranging from 0 to 10 with N = 100 molecules. Note that $N_e = 0$, implies there is no coupling and only the bare photon contributes to the spectra. (c) Error (with respect to $N_e = 10$) in the spectra as a function of the number of included electronic states.

Further Discussions and Additional Results

Further Discussions of Fig. 2 of Main text.

There are two distinct types of "dark states" that arise in this work: (i) the usual dark states that are referred to in calculations with $N > 1$ and (ii) the dark states that arise in calculations with $N_{\rm e} > 1$ (even for the case of $N = 1$). In both cases, when considering the disorders (geometry fluctuations), these states become partially bright[21] (*i.e.*, gain finite photonic character) with a finite light-matter coupling A_0. However, only the case (ii) states are activated *in the absence of molecular disorder*, while the case (i) states are dark by symmetry, without disorder, for all coupling strengths A_0. In general, all states have some photonic character due to the diagonal (transition energy) and off-diagonal (transition dipole) disorder present in the Hamiltonian. In both cases, the accumulation of dark-state photonic character acts as a primary broadening mechanism for the polaritonic spectral bands. In the present system, since there are no electronic states below the cavity frequency, the lower polariton is unaffected, while the upper polariton's spectral feature is broadened.

As an aside, we find that the intensity of the spectra at the midpoint between the upper and lower peaks decreases both in simulation and experiment as the collective light-matter coupling increases due to the decrease in overlap of the two spectral features. For the theoretical simulations, comparing $N = 1$ and $N = 100$, both with $N_{\rm e} = 50$ (green curves in Fig. 3), we note a slightly increased intensity at the midpoint for $N_{\rm e} = 50$ compared to $N = 1$, due to the additional states with partial photonic character. Furthermore, we note that the lower polariton is slightly thinned for the $N = 100$ compared to the $N = 1$ case. Finally, it is important to point out that, even without thermal disorder, including the additional electronically excited states already introduces non-negligible features due to the additional interactions with the upper polariton branch.

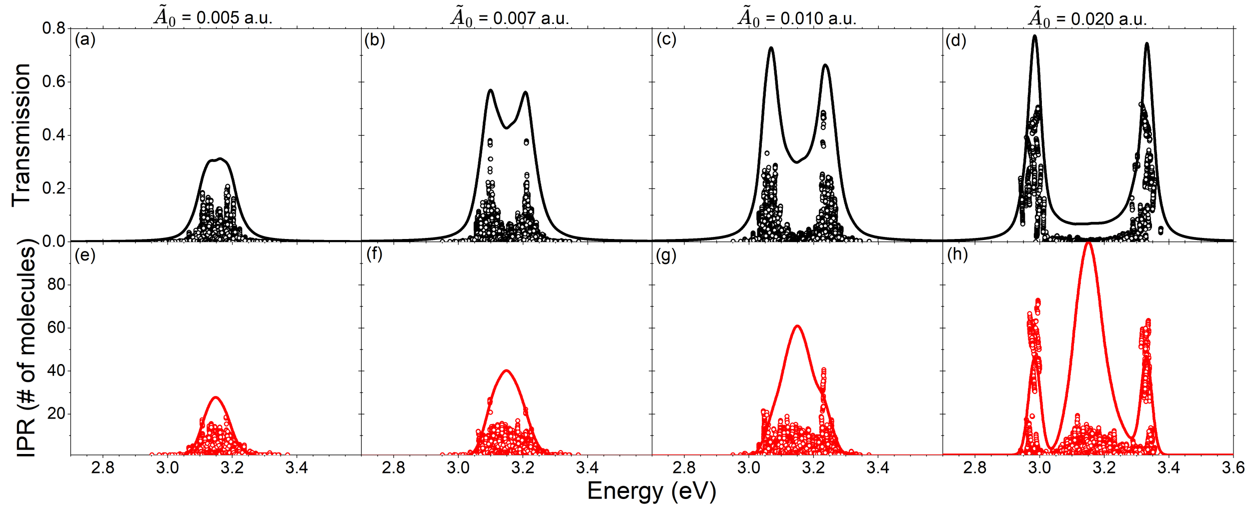

Figure S3: (a,b,c,d) Transmission spectra and (e,f,g,h) inverse participation ratio (IPR) at varying collective light-matter coupling strengths $\mathcal{A}_N = 0.005$ (a,e), 0.007 (b,f), 0.010 (c,g), and 0.020 (d,h) a.u.. In all panels, the cavity frequency $\omega_c = 3.154$ eV and $N = 100$ molecules each with $N_e = 1$ electronic states per molecule. The continuous curves represent the convolution of a finite-width ($\sigma = 15$ meV) Lorentzian curve for each snapshot and are averaged over 901 snapshots.

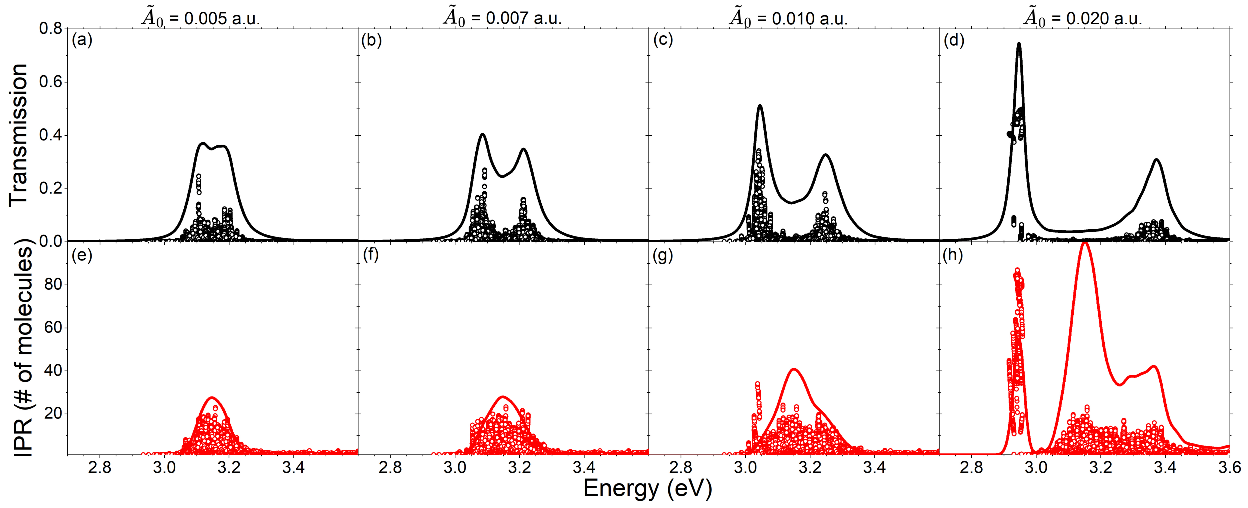

Figure S4: (a,b,c,d) Transmission spectra and (e,f,g,h) inverse participation ratio (IPR) at varying collective light-matter coupling strengths $\mathcal{A}_N = 0.005$ (a,e), 0.007 (b,f), 0.10 (c,g), and 0.020 (d,h) a.u.. In all panels, the cavity frequency $\omega_c = 3.154$ eV and $N = 100$ molecules each with $N_e = 50$ electronic states per molecule. The continuous curves represent the convolution of a finite-width ($\sigma = 15$ meV) Lorentzian curve for each snapshot and are averaged over 901 snapshots.

Further Discussions of Figs. 3 of Main text.

Figs. S3 and S4 present additional light-matter coupling strength results related to Fig. 3 in the **Main Text** for the $N_\mathrm{e} = 1$ (Fig. S3) and $N_\mathrm{e} = 50$ (Fig. S4) cases. Specifically, the additional results provide transmission spectra (top row, a-d) and IPR (bottom rows, e-h) for two smaller Rabi splittings Ω_R simulated with collective light-matter coupling streagnths $\mathcal{A}_N = 0.005$ a.u. (a,e) and $\mathcal{A}_N = 0.007$ a.u. (b,f). The other two coupling strengths, $\mathcal{A}_N = 0.010$ a.u. (c,g) and $\mathcal{A}_N = 0.020$ a.u. (d,h) are the same data as shown in Fig. 3 in the **Main Text**.

At weak coupling, $\mathcal{A}_N = 0.005$ a.u. (a,e) the upper and lower polaritonic bands are indistinguishable in both the $N_\mathrm{e} = 1$ and 50 cases. However, the individual intensities of the transmission spectra (see Eq. 7) are already showing upper and lower polaritonic character. For the next strongest coupling, $\mathcal{A}_N = 0.007$ a.u. (b,f), the upper and lower polaritonic peaks have emerged in both N_e cases. At this point, for $\mathcal{A}_N = 0.007$ a.u., it is already evident that including more than one electronic state provides a substantial asymmetry to the spectral intensities of the upper and lower polaritons. Even in $\mathcal{A}_N = 0.005$ a.u., the individual intensities shows this asymmetry for the $N_\mathrm{e} = 50$ case (Fig. S4a) and not for the $N_\mathrm{e} = 1$ case (Fig. S3a).

The IPR for the two additional weak coupling cases shows that the polaritonins are largely localized to less than 20% of the molecules, regardless of the number of electronic states N_e used in the Hamiltonian. At larger coupling strengths (g,h), it is clear that the polaritonic delocalization takes effect, as discussed thoroughly in the **Main Text**.

Orientational disorder in Molecular Polariton.

We now consider the orientational disorder of molecules having an isotropic orientation of molecules with respect to the cavity polarization $\mathbf{e}$, such that for molecule n

$$\hat{\boldsymbol{\mu}}_n \cdot \hat{\mathbf{e}} = \sin\theta\cos\phi\ \hat{\boldsymbol{\mu}}_n \cdot \mathbf{X} + \sin\theta\sin\phi\ \hat{\boldsymbol{\mu}}_n \cdot \mathbf{Y} + \cos\theta\ \hat{\boldsymbol{\mu}} \cdot \mathbf{Z}. \tag{S5}$$

where $\hat{\boldsymbol{\mu}}_n \cdot \mathbf{X}$, $\hat{\boldsymbol{\mu}}_n \cdot \mathbf{Y}$, $\hat{\boldsymbol{\mu}}_n \cdot \mathbf{Z}$ are the dipole operator $\hat{\boldsymbol{\mu}}_n$ projected along the $\mathbf{X}$, $\mathbf{Y}$ and $\mathbf{Z}$ directions and $\theta \in [0, \pi)$ and $\phi \in [0, 2\pi)$ were uniformly sampled. See **Theoretical Methods** for more details.

Fig. S5 shows how additional angular disorder affects the transmission spectra (Fig. S5a,b) and

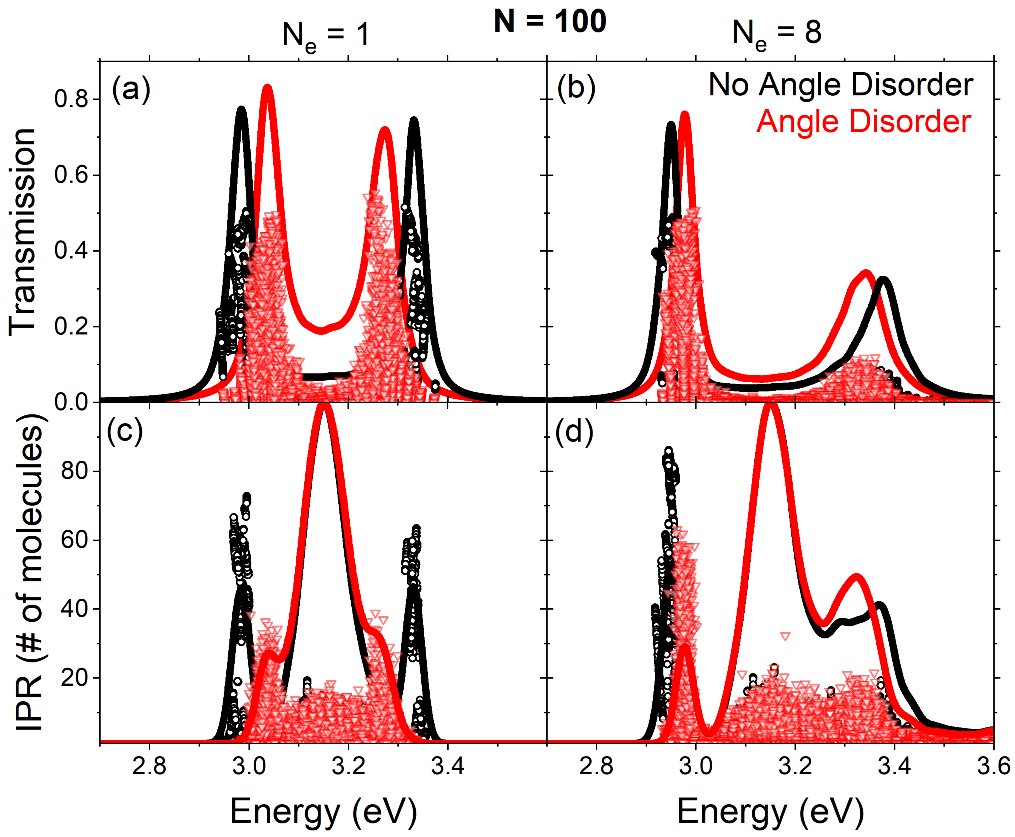

Figure S5: (a,b) Transmission spectra and (c,d) inverse participation ratio (IPR) at collective light-matter coupling strength $\mathcal{A}_N = 0.02$ a.u. without (black) and with (red) angular disorder between the molecular dipole orientation direction and the cavity polarization direction. The energetic/thermal disorders are present in all cases. The cavity frequency $\omega_c = 3.154$ eV and $N = 100$ molecules. Panels (a,c) have $N_e = 1$ electronic states per molecule, while panels (b,d) have $N_e = 8$. The IPR "spectra" [solid black and red curves in panels (c,d)] are interpreted as a delocalization (or IPR) density and are primarily used only for a qualitative guide for the eye (see Eq. S3 in **Supporting Information**).

IPR (Fig. S5c,d) for the cases of $N_e = 1$ (Fig. S5a,c) and $N_e = 8$ (Fig. S5b,d), all with $N = 100$ at collective light-matter coupling $\mathcal{A}_N = 0.020$ a.u. The black curves represent similar values. data and qualitatively identical features as shown in Fig. 3, which includes thermal disorder effects due to the geometry-induced energy and dipole matrix elements fluctuations. The red curves have the same parameters as the black, except they are additionally averaged over 10 random sets of angles (θ, ϕ) for each molecule. In total, there are 901 averages for the non-angle-disordered results and $901 \times 10 = 9010$ averages for the angle-disordered results.

We find that the angle disorder introduces a contraction of the effective Rabi splitting Ω_R.

This result is expected from previous theoretical work based on the Tavis-Cummings model,[22–24] which predicts that the Rabi splitting will be reduced to a $\Omega_R/\sqrt{3} \approx 0.577$. For the $N_e = 1$ case (Fig. S5a), we find close agreement with the analytic result. On the other hand, when considering $N_e = 8$, due to the asymmetry of the electronic states, the angular disorder introduces only a slight change to the upper and lower polaritonic spectral bands, while introducing additional broadening in the upper polariton feature, indicating that the angular sampling reduces the amount of B_{XY} character, on average, favoring the higher-energy excitonic states in the wavefunction expansion of the UP feature.

The delocalization of the polaritonic states and dark states is also affected by the orientational disorder. In both cases, $N_e = 1$ and $N_e = 8$, the middle polaritonic/dark states are already semi-delocalized across the molecular system due to the thermal disorder. For the $N_e = 1$ case (Fig. S5c), the magnitude of the IPR decreases with the addition of angular disorder (black to red), in parallel with the reduction in Rabi splitting. For the $N_e = 8$ case (Fig. S5d), the angular disorder has a smaller effect compared to the $N_e = 1$ case. The orientational disorder's effect reduces the energetic splitting between the polariton peaks and therefore increases the spectral overlap between the upper and lower polaritonic bands with the manifold of dark states.

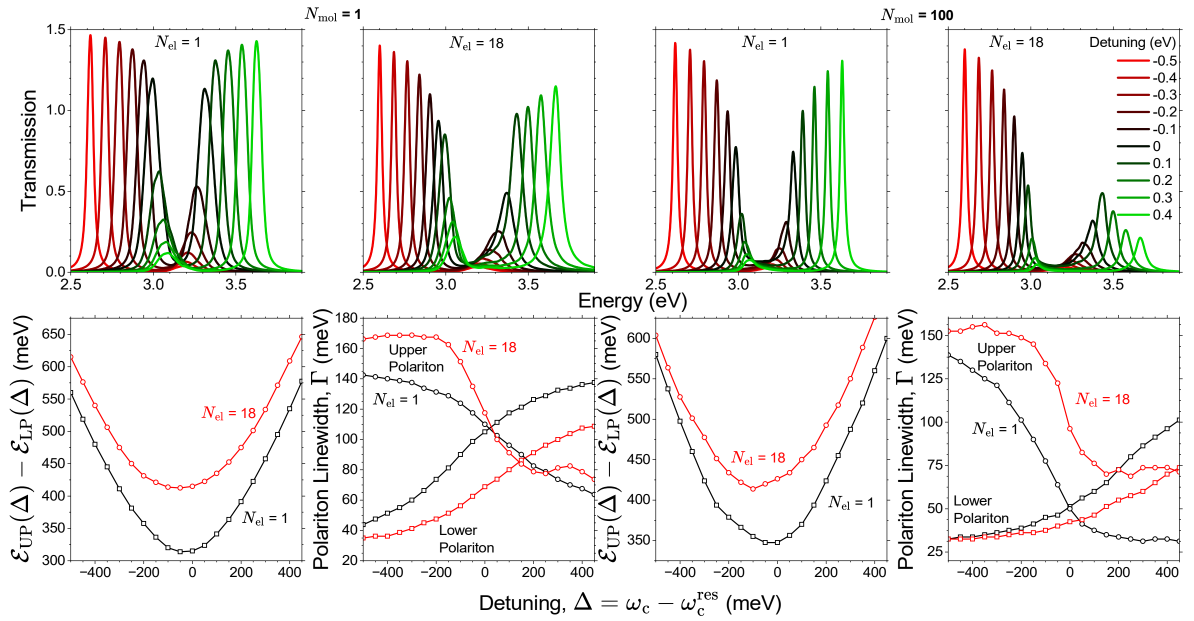

Figure S6: (top row) Transmission spectra as a function of the cavity detuning $\Delta = \omega_{\rm c} - \omega_{\rm c}^{\rm res}$, where $\omega_{\rm c}^{\rm res} = 3.154$ eV is the resonant cavity frequency used in all previous figures, for various Hamiltonians (columns). Rabi splitting $\Omega_{\rm R}$ (bottom row, first and third columns) and polariton broadening Γ (bottom row, second and fourth columns) as functions of the cavity detuning Δ. For all panels, the collective light-matter coupling strength is $\mathcal{A}_N = 0.020$ a.u..

Additional Results for Fig. 4 of the main text.

Fig. S6 presents additional data for Fig. 4 in the **Main Text**. The top row shows spectra for all detununings and for all four types light-matter Hamiltonians: $N = 1$ & $N_{\rm e} = 1$ (first column), $N = 1$ & $N_{\rm e} = 18$ (second column), $N = 100$ & $N_{\rm e} = 1$ (third column), $N = 100$ & $N_{\rm e} = 18$ (fourth column). The bottom row presents the Rabi splitting (first and third columns) and the polaritonic broadening (second and fourth columns) for each of the four types of Hamiltonians. All data presented here are for the largest collective light-matter coupling strength $\mathcal{A}_N = 0.020$ a.u. All of the data in the bottom row, except for the $N = 1$ & $N_{\rm e} = 18$ Hamiltonian results, are reproduced from Fig. 4 in the **Main Text**. The spectra for the $N = 1$ & $N_{\rm e} = 1$, $N = 1$ & $N_{\rm e} = 18$, and $N = 100$ & $N_{\rm e} = 1$ cases in the top row are not shown in Fig. 4 in the **Main Text**.

Similar to Fig. 4d in the **Main Text**, Fig S7 shows the polariton linewidth Γ as a function of exciton fraction $|X|^2$ for all Hamiltonians (colors) as well as for both the upper (filled symbols) and lower (open symbols) polaritonic spectral features. Note that the broadening parameter for the spectra was set to $\sigma = 15$ meV, accounting for the intrinsic polariton broadening (*i.e.* photon loss)

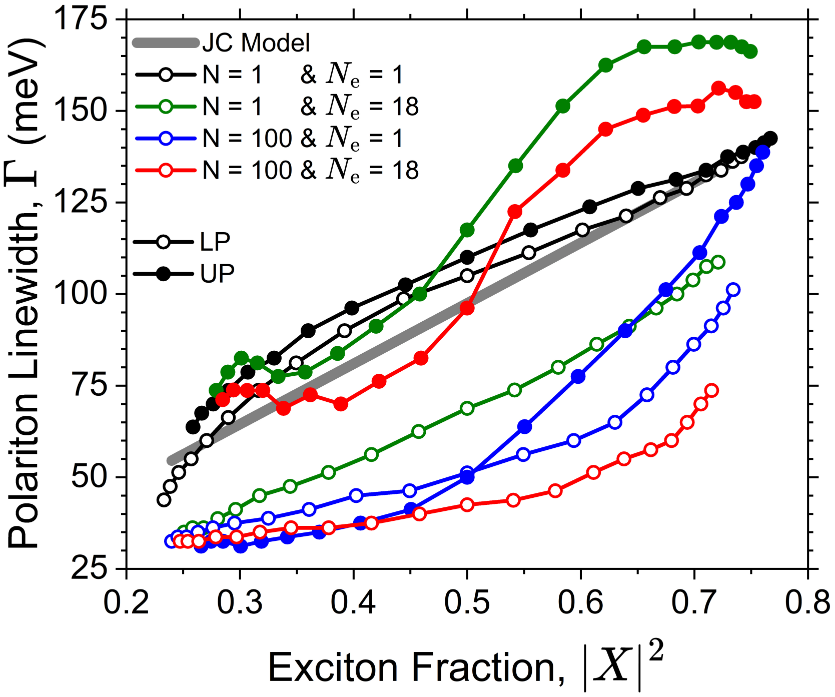

Figure S7: Polariton linewidth Γ as a function of exciton fraction $|X|^2$ for all Hamiltonians (colors) as well as for both the upper (filled symbols) and lower (open symbols) polaritonic spectral features. Note that the broadening parameter for the spectra was set to $\sigma = 15$ meV, accounting for the intrinsic polariton broadening (*i.e.* photon loss) as well as for the dynamical exciton disorder. The static exciton disorder is accounted for through thermal sampling.

as well as for the dynamical exciton disorder. The static exciton disorder is accounted for through thermal sampling.

Table S1: Energies (E) and oscillator strengths (f) for the first 50 electronic excitations for a single nuclear snapshot.

State	E (eV)	f	State	E (eV)	f
S1	2.31	0.15	S26	4.25	0.80
S2	2.32	0.12	S27	4.26	0.47
S3	3.17	9.73	S28	4.29	2.73
S4	3.21	21.04	S29	4.31	1.15
S5	3.31	23.90	S30	4.32	0.30
S6	3.43	12.92	S31	4.34	0.08
S7	3.67	0.11	S32	4.36	0.67
S8	3.76	1.08	S33	4.36	0.12
S9	3.78	0.89	S34	4.37	0.03
S10	3.87	0.18	S35	4.38	0.28
S11	3.91	0.25	S36	4.39	0.03
S12	3.93	0.27	S37	4.41	0.03
S13	3.97	0.11	S38	4.42	0.39
S14	3.99	0.44	S39	4.43	0.13
S15	4.03	0.05	S40	4.44	0.09
S16	4.05	0.03	S41	4.49	0.12
S17	4.07	0.17	S42	4.50	0.08
S18	4.10	0.23	S43	4.51	0.02
S19	4.13	0.68	S44	4.53	0.34
S20	4.14	0.05	S45	4.54	0.57
S21	4.17	1.16	S46	4.56	0.18
S22	4.19	0.01	S47	4.56	0.33
S23	4.20	0.10	S48	4.60	0.02
S24	4.21	0.34	S49	4.61	0.68
S25	4.22	0.16	S50	4.71	0.34

References

(1) Weight, B. M.; Mandal, A.; Huo, P. Semiclassical Quantum Dynamics (SQD). 2023; `https://github.com/bradenmweight/SQD`, original-date: 2023-01-15T22:28:05Z.

(2) Dewar, M. J. S.; Zoebisch, E. G.; Healy, E. F.; Stewart, J. J. P. Development and use of quantum mechanical molecular models. 76. AM1: a new general purpose quantum mechanical molecular model. *Journal of the American Chemical Society* **1985**, *107*, 3902–3909.

(3) Frisch, M. J. et al. Gaussian 16. 2016; Gaussian Inc. Wallingford CT.

(4) Lu, T.; Chen, F. Multiwfn: A multifunctional wavefunction analyzer. *Journal of Computational Chemistry* **2012**, *33*, 580–592.

(5) Weight, B. M.; Krauss, T. D.; Huo, P. Investigating Molecular Exciton Polaritons Using Ab Initio Cavity Quantum Electrodynamics. *The Journal of Physical Chemistry Letters* **2023**, *14*, 5901–5913.

(6) Weight, B. M.; Weix, D. J.; Tonzetich, Z. J.; Krauss, T. D.; Huo, P. Cavity Quantum Electrodynamics Enables para- and ortho-Selective Electrophilic Bromination of Nitrobenzene. *Journal of the American Chemical Society* **2024**, *146*, 16184–16193.

(7) Wang, J.; Weight, B. M.; Huo, P. Investigating Cavity Quantum Electrodynamics-Enabled Endo/Exo-Selectivities in a Diels–Alder Reaction. *The Journal of Physical Chemistry A* **2025**, *129*, 5458–5468.

(8) Schäfer, C.; Ruggenthaler, M.; Rokaj, V.; Rubio, A. Relevance of the Quadratic Diamagnetic and Self-Polarization Terms in Cavity Quantum Electrodynamics. *ACS Photonics* **2020**, *7*, 975–990.

(9) Rokaj, V.; Welakuh, D. M.; Ruggenthaler, M.; Rubio, A. Light–matter interaction in the long-wavelength limit: no ground-state without dipole self-energy. *J. Phys. B: At. Mol. Opt. Phys.* **2018**, *51*, 034005.

(10) Foley, J. J., IV; McTague, J. F.; DePrince, A. E., III Ab initio methods for polariton chemistry. *Chem. Phys. Rev.* **2023**, *4*, 041301.

(11) Bauman, N. et al. Perspective on Many-Body Methods for Molecular Polaritonic Systems. *J. Chem. Theory Comput.* **2025**, *21*, 10035–10067.

(12) Liebenthal, M. D.; Vu, N.; DePrince, A. E. I. Assessing the Effects of Orbital Relaxation and the Coherent-State Transformation in Quantum Electrodynamics Density Functional and Coupled-Cluster Theories. *J. Phys. Chem. A* **2023**, *127*, 5264–5275.

(13) Li, X.; Zhang, Y. First-principles molecular quantum electrodynamics theory at all coupling strengths. 2023; http://arxiv.org/abs/2310.18228, arXiv:2310.18228 [physics].

(14) Mazin, I.; Zhang, Y. Light-Matter Hybridization and Entanglement from the First-Principles. 2024; http://arxiv.org/abs/2411.15022, arXiv:2411.15022 [quant-ph].

(15) Cui, Z.-H.; Mandal, A.; Reichman, D. R. Variational Lang–Firsov Approach Plus Møller–Plesset Perturbation Theory with Applications to Ab Initio Polariton Chemistry. *Journal of Chemical Theory and Computation* **2024**, *20*, 1143–1156.

(16) Haugland, T. S.; Ronca, E.; Kjønstad, E. F.; Rubio, A.; Koch, H. Coupled Cluster Theory for Molecular Polaritons: Changing Ground and Excited States. *Physical Review X* **2020**, *10*, 041043.

(17) Weight, B. M.; Zhang, Y. Auxiliary Field Quantum Monte Carlo for Electron-Photon Correlation. 2025; http://arxiv.org/abs/2505.16021.

(18) Weight, B. M.; Tretiak, S.; Zhang, Y. Diffusion quantum Monte Carlo approach to the polaritonic ground state. *Physical Review A* **2024**, *109*, 032804.

(19) Weight, B. M.; Li, X.; Zhang, Y. Theory and modeling of light-matter interactions in chemistry: current and future. *Physical Chemistry Chemical Physics* **2023**, *25*, 31554–31577.

(20) Avramenko, A. G.; Rury, A. S. Local molecular probes of ultrafast relaxation channels in strongly coupled metalloporphyrin-cavity systems. *The Journal of Chemical Physics* **2021**, *155*, 064702.

(21) Qiu, L.; Mandal, A.; Morshed, O.; Meidenbauer, M. T.; Girten, W.; Huo, P.; Vamivakas, A. N.; Krauss, T. D. Molecular Polaritons Generated from Strong Coupling between CdSe Nanoplatelets and a Dielectric Optical Cavity. *The Journal of Physical Chemistry Letters* **2021**, *12*, 5030–5038.

(22) Houdré, R.; Stanley, R. P.; Ilegems, M. Vacuum-field Rabi splitting in the presence of inhomogeneous broadening: Resolution of a homogeneous linewidth in an inhomogeneously broadened system. *Phys. Rev. A* **1996**, *53*, 2711–2715.

(23) Mondal, M. E.; Vamivakas, A. N.; Cundiff, S. T.; Krauss, T. D.; Huo, P. Polariton spectra under the collective coupling regime. I. Efficient simulation of linear spectra and quantum dynamics. *J. Chem. Phys.* **2025**, *162*, 014114.

(24) Lai, Y.; Ying, W.; Krauss, T.; Huo, P. Analytic Rate Theory of Polariton Relaxation that Explains Long Polaritonic Lifetime. *J. Chem. Phys.* **2026**, *164*, 024103.
